# Mechanistic insights into dopaminergic and serotonergic neurotransmission – concerted interactions with helices 5 and 6 drive the functional outcome†

**DOI:** 10.1039/d1sc00749a

**Published:** 2021-07-02

**Authors:** Tomasz Maciej Stepniewski, Arturo Mancini, Richard Ågren, Mariona Torrens-Fontanals, Meriem Semache, Michel Bouvier, Kristoffer Sahlholm, Billy Breton, Jana Selent

**Affiliations:** Research Programme on Biomedical Informatics (GRIB), Hospital del Mar Medical Research Institute (IMIM) – Pompeu Fabra University (UPF) Dr Aiguader 88 Barcelona E-08003 Spain jana.selent@upf.edu; InterAx Biotech AG, PARK InnovAARE 5234 Villigen Switzerland; Domain Therapeutics NA Inc 7171 Frederick-Banting Saint-Laurent (QC) H4S 1Z9 Canada amancini@domaintherapeutics.com; Department of Neuroscience, Karolinska Institute Stockholm Sweden; Department of Biochemistry and Molecular Medicine, Université de Montréal Montreal QC H3C 3J7 Canada; Institute for Research in Immunology and Cancer (IRIC), Université de Montréal Montréal Québec H3T 1J4 Canada; Department of Integrative Medical Biology, Wallenberg Centre for Molecular Medicine, Umeå University 90187 Umeå Sweden

## Abstract

Brain functions rely on neurotransmitters that mediate communication between billions of neurons. Disruption of this communication can result in a plethora of psychiatric and neurological disorders. In this work, we combine molecular dynamics simulations, live-cell biosensor and electrophysiological assays to investigate the action of the neurotransmitter dopamine at the dopaminergic D_2_ receptor (D_2_R). The study of dopamine and closely related chemical probes reveals how neurotransmitter binding translates into the activation of distinct subsets of D_2_R effectors (*i.e.*: G_i2_, G_oB_, G_z_ and β-arrestin 2). Ligand interactions with key residues in TM5 (S5.42) and TM6 (H6.55) in the D_2_R binding pocket yield a dopamine-like coupling signature, whereas exclusive TM5 interaction is typically linked to preferential G protein coupling (in particular G_oB_) over β-arrestin. Further experiments for serotonin receptors indicate that the reported molecular mechanism is shared by other monoaminergic neurotransmitter receptors. Ultimately, our study highlights how sequence variation in position 6.55 is used by nature to fine-tune β-arrestin recruitment and in turn receptor signaling and internalization of neurotransmitter receptors.

## Abstract 2

Neurotransmitter contacts within the receptor binding site differentially contribute to the overall functional response: transmembrane helix (TM) 5 contacts promote G protein coupling whereas concerted TM5–TM6 contacts enhance β-arrestin recruitment.

## Graphical abstract

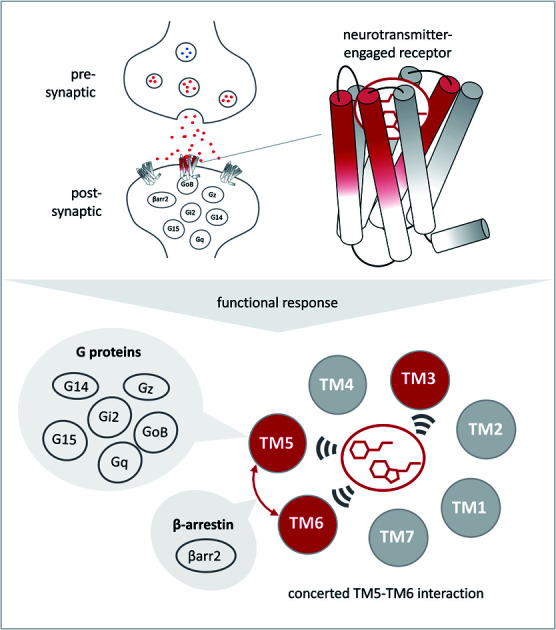

## Introduction

Neurotransmitters are chemical messengers that mediate communication between billions of neurons within an enormous network that constitutes the central nervous system (CNS). Disruptions in the regulation of this system are known to result in numerous disorders, including depression, psychosis, bipolar disorder, general anxiety disorder and Parkinson's disease.^1^ Neurotransmitters mediate their effects *via* numerous G protein coupled receptors (GPCRs), which comprise the largest family of human cell surface receptors.^2^ Herein, we focus on the dopamine D_2_ receptor (D_2_R) and its endogenous agonist dopamine – a neurotransmitter with a catecholamine scaffold^3^ that is also common to other signaling molecules (*e.g.*, adrenaline or noradrenaline). In response to dopamine, the D_2_R is known to signal through both G protein (*i.e.*, Gi_1-3_, Go_A-B_, G_z_) and β-arrestin (βarr2) signaling pathways. This coupling profile modulates important processes in the brain related to memory, learning, attention, mood and movement. The observation that some ligands can preferentially engage one pathway over others has led to the concept of signaling bias or functional selectivity.^4,5^ This groundwork has initiated the quest for more efficient and safer CNS-targeting drugs able to preferentially engage therapeutically relevant pathways over those responsible for deleterious side-effects.^6–8^ Despite first insights,^9,10^ the rational design of drugs with a desired signaling profile remains a significant challenge as it is difficult to pinpoint ligand–receptor interactions responsible for a specific coupling profile. Even subtle changes in ligand–receptor interactions can result in a dramatic change of the signaling profile.^11^ We envisage that disclosing the molecular link between dopamine binding and the induced D_2_R coupling profile to different intracellular partners can contribute to a wider understanding of neurotransmission. Beyond, it can also guide the development of ligands with a tailored coupling profile for this receptor and other aminergic GPCRs.

Throughout the last decades, several research groups have studied the binding and functional outcome for dopamine and its analogues.^12–15^ Unfortunately, the results are not always consistent between studies, likely due to inter-study differences in experimental setups. In addition, the atomistic resolution of how dopamine binding translates into the recruitment of distinct intracellular signaling proteins (*e.g.*, G_oB_, G_z_, G_i2_, βarr2) remains unclear despite the recently solved D_2_R in complex with the G_i_ protein.^16^ To address this knowledge gap, we carried out all-atom molecular dynamics simulation (classical and enhanced sampling techniques) accumulating ∼40 μs of simulation time. The power of enhanced sampling techniques for capturing biologically relevant events has been shown in previous studies.^17–19^ Here, we use this approach to construct the complete energetic binding landscape of dopamine and closely related signaling probes. The small size and low number of rotatable bonds of studied compounds allow for an exhaustive sampling of their binding. The signaling signature of each ligand was characterized by assessing receptor coupling to a selection of intracellular signaling proteins with relevance for neurotransmission (G_oB_, G_z_, G_i2_, βarr2) using live-cell BRET-based biosensors. Ultimately, this approach allowed us to detect a mechanism shared by several aminergic GPCRs with relevance for neurotransmission.

## Results

### Simultaneous TM5 and TM6 contacts contribute to the dopamine-like coupling outcome

**Fig. 1:**
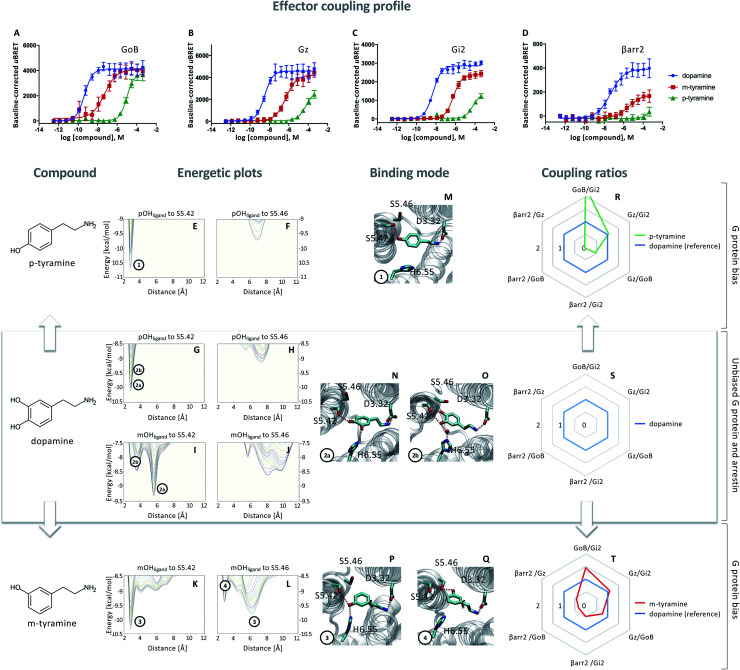
Coupling and binding profile of dopamine, *p*-tyramine and *m*-tyramine. The corresponding chemical structures are depicted on the left. (A–D) Concentration–response curves of dopamine (blue), *p*-tyramine (green) and *m*-tyramine (red)-induced coupling to G_oB_, G_z_, G_i2_ and β-arrestin 2 (βarr2) at the D_2_R. For corresponding pEC_50_ and *E*_max_ values see Table S3.† (E–L) Energetic plots of ligand binding obtained by metadynamics using as metrics the distance of the *m*- and/or *p*-OH groups to S5.42 and S5.46. An energetic well at ∼2.8 Å indicates a favorable distance for binding contacts with the corresponding residue. To ensure convergence of binding energetics, we monitored free energy profiles along simulation by plotting the profile every 20 000 deposited Gaussian (graphs shown in different colors). (M–Q) Representative structures of the binding mode corresponding to the energetic wells identified in the energetic plots. (R–T) Coupling ratios were approximated using the area under the curve (AUC) and its ratios for individual signaling effectors (*e.g.* βarr2 *vs.* G_z_, βarr2 *vs.* G_oB_*etc.*). To eliminate observational bias linked to differences within different biosensor assays (*e.g.* βarr2 *vs.* G_i_), we use dopamine as an internal standard for analyzing the AUCs. The coupling profile of the reference compound dopamine is denoted by a coupling ratio of 1 for all pathway combinations and highlighted in all plots as a blue line. Preferential or disfavored coupling (*vs.* dopamine) are indicated by ratios > 1 or < 1, respectively. Concentration–response curves were generated using data obtained from 3 independent experiments. Baseline *u*BRET values were subtracted from concentration–response curves.

In a first step, we evaluated the ability of dopamine to promote the engagement of different G_i_ proteins (G_oB_, G_z_, G_i2_) and βarr2 by D_2_R using live-cell BRET-based biosensors. The BRET-based assay confirms robust coupling of the D_2_R to all tested intracellular effector proteins upon dopamine binding (Fig. 1A–D, blue lines). Differences can be observed in ligand potency whereas the highest potency is found for G_oB_ followed by G_z_, G_i2_ and βarr2 (Table S3A†).

Next, we probed the general binding mode of dopamine using classical unbiased molecular dynamics simulations. We were able to reproduce known binding characteristics, including polar contacts of the *meta* (*m*-OH) and the *para* (*p*-OH) hydroxyl groups of dopamine to TM5 and TM6 (Fig. S1†) which are in agreement with site-directed mutagenesis.^12–15,20,21^ Despite the structural insights provided by different mutational studies, specific contributions of the individual *p*- and *m*-OH groups to the binding and functional outcome of dopamine or its analogues remain unclear (Table S2A and B†). To address this question, we used metadynamics to construct the energetic map of dopamine binding, focusing on its *p*-/*m*-OH groups and their preferred binding contacts to residues in TM5 (S5.42 and S5.46) (Fig. 1).

A binding contact can be appreciated as an energetic well at approximately 2.8 Å distance between the *p*-/*m*-OH group and the polar residues (S5.42 or S5.46) in TM5. For the *p*-OH group, we find one binding peak for S5.42 (Fig. 1G) whereas no interaction peak is found for S5.46 (Fig. 1H). Note that the binding peak for S5.42 corresponds to the two binding modes 2a and 2b (Fig. 1N–O). These different binding modes are the results of different orientations of the *m*-OH group which becomes evident when plotting the binding preference of the *m*-OH group relative to S5.42 (Fig. 1I, separated peaks 2a and 2b). Despite their difference in binding, both modes (Fig. 1N–O) allow for simultaneous interaction with TM5 (*via p*-OH) and TM6 (*via m*-OH). It is worth noting that TM6 interaction can be direct (peak 2a) or indirect *via* a water molecule (peak 2b), as also suggested by unbiased simulation (Fig. S1†). It is tempting to speculate that simultaneous interaction between TM5 and 6 contributes to the dopamine coupling profile involving G_oB_, G_i2_, G_z_ and βarr2 engagement.

### Exclusive TM5 interaction results in preferential G protein over βarr2 coupling

To dissect the contribution of *p*-OH and *m*-OH for the D_2_R coupling outcome, we studied *p*-tyramine – a molecule that only exposes the *p*-OH group (Fig. 1, left side). Not surprisingly, energetic maps indicate that the *p*-OH group interacts exclusively with S5.42 in TM5 (Fig. 1E and F) similar to dopamine (Fig. 1G and H). Due to the lack of *m*-OH, no simultaneous interactions are formed with TM5 and 6 (Fig. 1M *vs*. 1N and O). Importantly, this structural difference translates into a substantial alteration of the coupling signature as seen in the corresponding concentration–response curves (Fig. 1A–D, green lines). We observe a reduction in potency and efficacy for all effector proteins compared to dopamine, with G_oB_ being least affected and βarr2 recruitment being almost entirely eliminated. Preferential G_oB_ coupling can be approximated when comparing the area under the curve (AUC) of the concentration–response curves which captures changes in both potency (EC_50_) and efficacy (*E*_max_). Calculating AUC-based coupling ratios (*e.g.* AUC_βarr2_*vs.* AUC_Gz_, AUC_βarr2_*vs.* AUC_GoB_, *etc.*) for *p*-tyramine corroborates a strong coupling preference of G_oB_ over βarr2 compared to the reference compound dopamine (Fig. 1R, blue: dopamine, green: *p*-tyramine). Note that a similar preferential coupling tendency is found for G_i2_ and G_z_ over βarr2. This finding suggests that exclusive TM5 interaction favors G protein recruitment, whereas additional interactions with TM6 *via* the *m*-OH promote the engagement of βarr2.

### Co-existence of two binding modes with different coupling signatures

Our present data suggest that m-OH interaction with TM6 promotes βarr2 coupling (*p*-tyramine *vs.* dopamine, Fig. 1A–D). To further test this hypothesis, we studied *m*-tyramine, a molecule that only carries a *m*-OH group, with the expectation that this compound should recruit βarr2 signaling to a similar extent as dopamine. Surprisingly, βarr2 was only partially engaged as seen in the concentration–response curves (Fig. 1D, red line) and corresponding AUC-based coupling ratios (Fig. 1T). Analyzing the binding of *m*-tyramine to the D_2_R, however, indicates the co-existence of two different binding modes. One binding mode is characterized by a binding peak to S5.42 (Fig. 1K). The corresponding state (Fig. 1P) allows for simultaneous interaction with S5.42 in TM5 as well as H6.55 in TM6 similar to dopamine. The second binding mode (peak 4 in Fig. 1L) involves a rotation of the aromatic ring directing its *m*-OH group to the bottom of the binding pocket. Such a structural constellation allows only for TM5 contacts (Fig. 1Q) which corresponds to a *p*-tyramine like binding mode (Fig. 1M). The co-existence of these two binding modes most likely explains the coupling outcome that is intermediate for all the tested effector proteins (Fig. 1A–D). According to the energetic plots, the energetic barrier between both binding modes (Fig. 1L, peaks 3 and 4) is approximately 1.3 kcal mol^−1^ and allows for frequent interconversion which in fact is also observed in classical unbiased simulations (Fig. S3†). Such ligand rotation is not surprising and captured in several X-ray structures (Table S5†).

### Proof of concept using rigid signaling probes

**Fig. 2:**
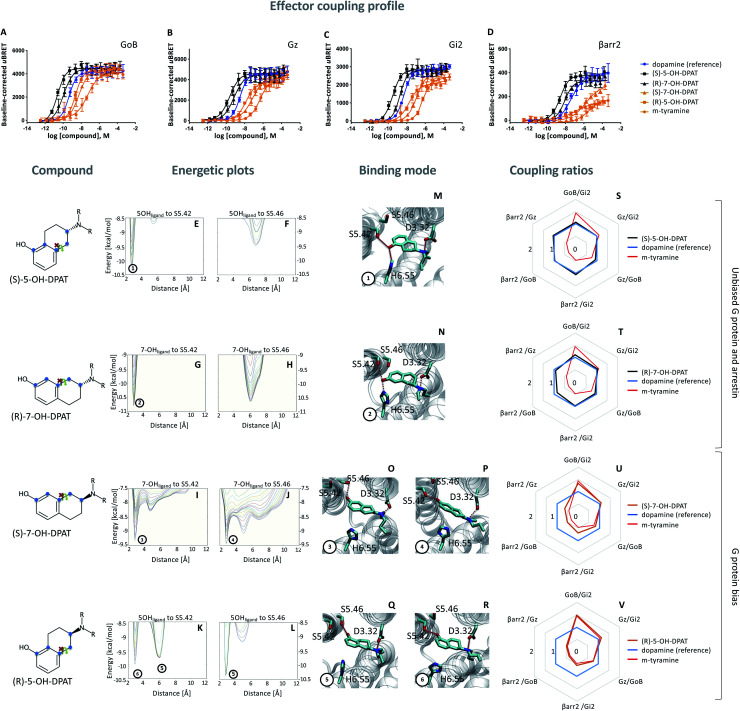
Coupling and binding profile of the (*R*)- and (*S*)-enantiomer of 5- and 7-OH-DPATs. The chemical structures of studied compounds are depicted on the left. Blue points mark the 5 carbon distance between OH group and amine group. (A–D) Concentration–response curves for (*S*)-5-OH-DPAT (black), (*R*)-7-OH-DPAT (black), (*R*)-5-OH-DPAT (orange) and (*S*)-7-OH-DPAT (orange) and their induced coupling to G_oB_, G_z_, G_i2_ and β-arrestin 2 (βarr2) at the D_2_R. Dopamine (blue) and *m*-tyramine (orange) are plotted as reference ligand. For corresponding pEC_50_ and *E*_max_ values see Table S3.† (E–L) Energetic plots of ligand binding obtained by metadynamics using as metrics the distance of the *m*- and/or *p*-OH groups to S5.42 and S5.46. An energetic well at ∼2.8 Å indicates a favorable distance for binding contacts with the corresponding residue. To ensure convergence of binding energetics, we monitored free energy profiles along simulation by plotting the profile every 20 000 deposited Gaussian (graphs shown in different colors). (M–R) Representative structures of the binding mode corresponding to the energetic wells identified in the energetic plots. (S–V) Coupling ratios were approximated using the area under the curve (AUC) and its ratios for individual signaling effectors (*e.g.* βarr2 *vs.* G_z_, βarr2 *vs.* G_oB_*etc.*). To eliminate observational bias linked to differences within different biosensor assays (*e.g.* βarr2 *vs.* G_i_), we use dopamine as internal standard for analyzing the AUCs. The coupling profile of the reference compound dopamine is denoted by a coupling ratio of 1 for all pathway combinations and highlighted in all plots as a blue line. Preferential or disfavored coupling (*vs.* dopamine) are indicated by ratios > 1 or < 1, respectively. The coupling ratio of *m*-tyramine has been included for comparison. Concentration–response curves were generated using data obtained from 3 independent experiments. Baseline *u*BRET values were subtracted from concentration–response curves.

Based on the hypothesis that the coupling profile of *m*-tyramine (Fig. 1T) is the result of the co-existence of two binding modes, we predicted that impeding the rotation of the hydroxylated aromatic ring (thus locking the compound in one or the other state; Fig. 1P or Q), should yield different coupling outcomes driven either by simultaneous TM5/6 interaction or exclusive TM5 contacts. To test this hypothesis, we used the rigid S and R enantiomers of 7-hydroxy-2-(di-*n*-propylamino)tetralins (7-OH-DPAT).^22^ Similar to *m*-tyramine, the OH-group of 7-OH-DPATs is separated by 5 carbons from the amine group (Fig. 2, see chemical structures). However, a main difference is that the bond that links the aromatic ring with the amine group has no rotational freedom due to ring condensation. Consequently, *R* and *S* enantiomers should adopt only one *m*-tyramine-like binding mode at a time. The obtained energetic landscape of ligand binding (Fig. 2G) supports this and shows that (*R*)-7-OH-DPAT interacts with S5.42 *via* its OH group and simultaneously with H6.55 in TM6 (Fig. 2N). In contrast, the related *S* enantiomer binds in an inverted position directing its OH group towards the bottom of the binding pocket interacting either with S5.42 or S5.46 (Fig. 2I and J) and allowing only for TM5 contacts (Fig. 2O and P). This is due to steric requirements that are extensively described in the ESI (Fig. S4†). Interestingly, a similar tendency is found for 5-OH-DPATs, which preserve 5-carbons distance between the OH-group and the amine group. Whereas (*S*)-5OH-DPAT establishes simultaneous TM5/6 contacts (Fig. 2M), the (*R*)-5OH-DPAT enantiomer adopts an inverted position with exclusive TM5 interactions (Fig. 2Q and R). Altogether, our simulation data indicate that by blocking the rotational freedom of *m*-tyramine, we are able to favor one binding mode at a time either with exclusive TM5 or simultaneous TM5/TM6 contacts.

As predicted, we found that different contact signatures of DPATs translate into distinct coupling profiles. Similar to dopamine (blue curve, Fig. 2A–D), DPATs with simultaneous TM5/TM6 contacts (black curves, Fig. 2A–D) maintain high potency and efficacy in engaging G_oB_, G_i2_, G_z_ and βarr2. In contrast, DPATs with exclusive TM5 contacts (orange curves) lose potency and efficacy (in particular for βarr2) when compared to dopamine (blue curves) while maintaining highest potency/efficacy at G_oB_. This trend is also reflected by the AUC-based coupling ratios, which show a dopamine-like profile for DPATs with TM5/6 interaction (black *vs.* blue lines, Fig. 2S and T) and a G_oB_-shifted coupling profile for DPATs with exclusive TM5 contacts (orange *vs.* blue lines, Fig. 2U and V).

To further support coupling differences for DPATs, we calculated the bias factor using the operational model.^23,24^ Whereas DPATs with TM5/6 contacts show no significant bias between pathways (Table S4†), DPATs with exclusive TM5 interaction ((*S*)-7-OH-DPAT and (*R*)-5-OH-DPAT) reach a 12- and 274-fold bias of G_oB_ over βarr2, respectively (Table S4A†).

### Relevance of H6.55 for the coupling outcome

**Fig. 3:**
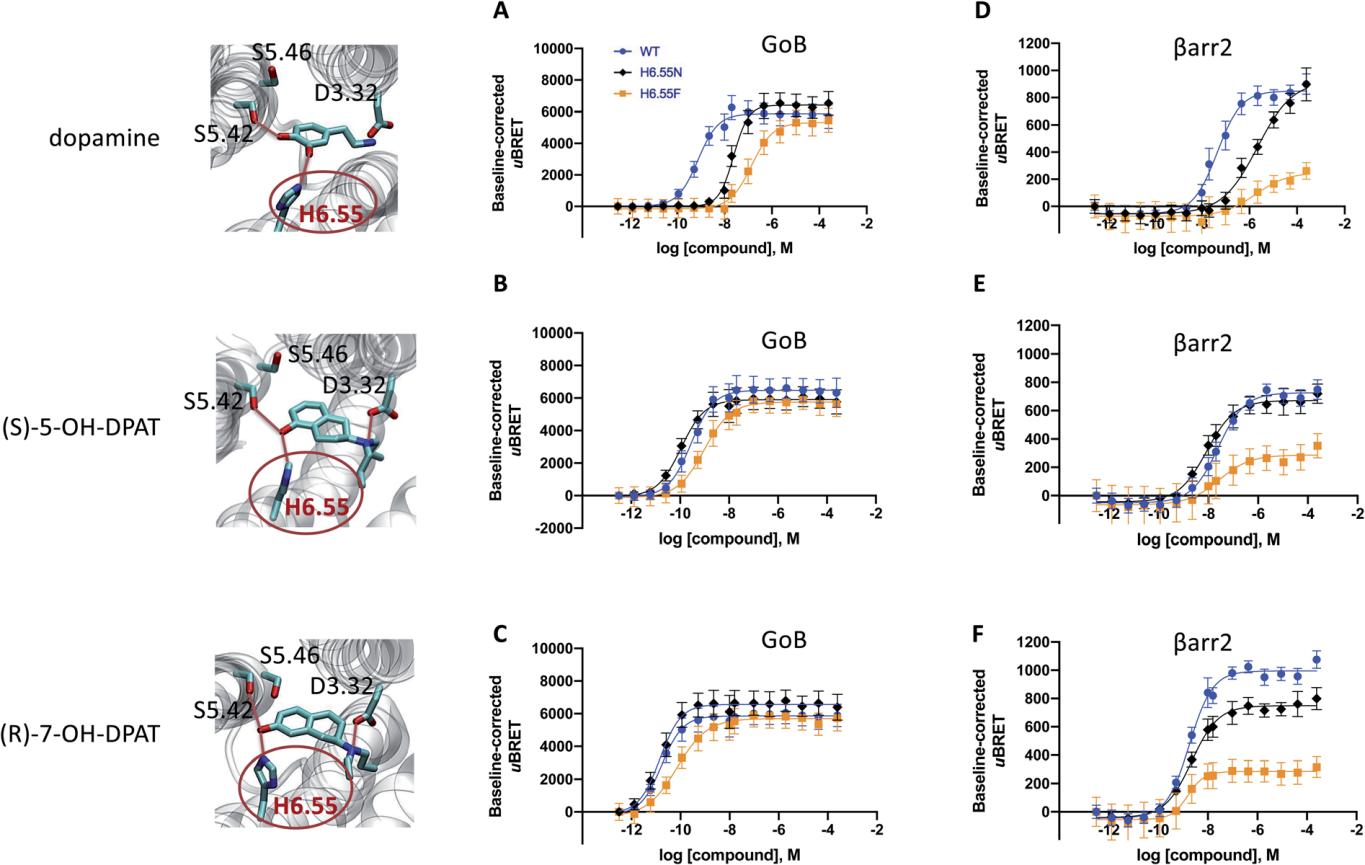
Site-directed mutation of position 6.55 and its impact on the coupling outcome at the D_2_R receptor. The coupling outcome of the D_2_R WT (blue line) compared to the H6.55N (black line) and H6.55F (orange line) mutants are plotted upon receptor stimulation with dopamine (A and D), (*S*)-5-OH-DPAT (B and E) and (*R*)-7-OH-DPAT (C and F). Concentration–response curves were obtained from 3 independent experiments. Baseline *u*BRET values were subtracted from concentration–response curves. For corresponding pEC_50_ and *E*_max_ values see Table S3C to H.†

Our simulation and signaling profiling experiments indicate that direct or indirect polar interactions with the key residue H6.55 in TM6 is a requirement for efficient coupling to βarr2 and less so for G proteins, in particular G_oB_. The relevance of ligand contacts with H6.55 for βarr2 engagement and in turn unbiased dopamine-like binding profiles is also reflected in contact heatmaps for ligand-D_2_R interactions at energetic minimum (Fig. S5†). To further assess the role of this residue, we introduced a phenylalanine into position 6.55 and evaluated its impact on G_oB_ and βarr2 coupling for dopamine as well as compounds with a dopamine-like coupling profile (*i.e.*, (*R*)-7-OH- and (*S*)-5-OH-DPAT). We selected a phenylalanine substitution as this is the residue most similar to histidine but lacking hydrogen donor/acceptor heteroatoms and thus effectively preventing polar interactions with position 6.55. Compared to the WT D_2_R, the H6.55F mutant displays a dramatic reduction in βarr2 recruitment (primarily in terms of efficacy, Fig. 3D–F) in response to dopamine and both DPATs whereas G_oB_ coupling is less impacted (Fig. 3A–C). This results in a preferential G_oB_ coupling over βarr2, an observation that is supported by the operational model^23,24^ with a bias (10^ΔΔlog(*τ*/*K*_A_)^) of ∼30 fold for dopamine and even ∼1000 fold for (*R*)-7-OH-DPAT or ≫1000 fold for (*S*)-5-OH-DPAT (Table S4B to D†). To further support the importance of polar contacts with H6.55, we introduced a polar residue (H6.55N) into position 6.55 as positive control. In fact, this mutant substantially recovers βarr2 coupling efficacies compared to the H6.55F mutant for all tested compounds underlining the relevance of polar contacts in this position for arrestin recruitment (Fig. 3D to F).

### Ligand coupling profiles correlate with time courses of agonist-evoked GIRK currents

**Fig. 4:**
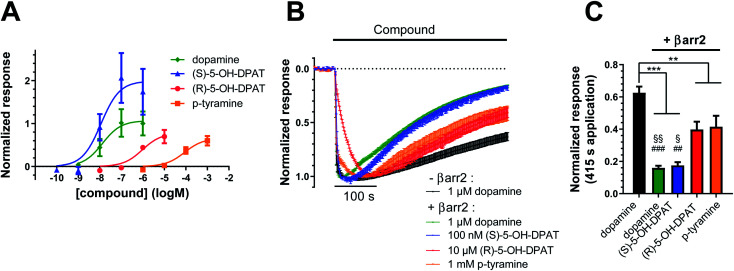
Potencies and desensitization rates of dopamine, (*S*)-, (*R*)-5-OH-DPAT, and *p*-tyramine at the D_2_R, as measured in the G protein-coupled inward rectifier potassium (GIRK) channel assay. (A) Concentration–response curves for GIRK activation by dopamine (pEC_50_ = 7.88 ± 0.40, *E*_max_ = 1.07 ± 0.20, *n* = 7), (*S*)-5-OH-DPAT (pEC_50_ = 7.94 ± 0.35, *E*_max_ = 1.99 ± 0.31, *n* = 4), (*R*)-5-OH-DPAT (pEC_50_ = 6.02 ± 0.27, *E*_max_ = 0.78 ± 0.12, *n* = 3) and *p*-tyramine (pEC_50_ = 4.06 ± 0.24, *E*_max_ = 0.66 ± 0.09, *n* = 5) in oocytes coexpressing D_2_R, GIRK1/4, RGS4, and βarr2. Agonist-induced responses were normalized to the maximal responses evoked by 1 μM dopamine in oocytes from the same batch. (B) Decay of agonist-induced GIRK currents during 415 s application of 1 μM dopamine to oocytes expressing D_2_R, GIRK1/4, and RGS4 without βarr2 is shown in black (*n* = 9). Coexpression of βarr2 induces varying degrees of desensitization following application of 1 μM dopamine (*n* = 11, green), 100 nM (*S*)-5-OH-DPAT (*n* = 7, blue), 10 μM (*R*)-5-OH-DPAT (*n* = 6, red) and 1 mM *p*-tyramine (*n* = 8, orange). Currents were normalized to their respective peak responses. Dotted line represents the agonist-independent baseline current level. (C) Summary statistics of residual currents after 415 s ligand applications (see B). Statistical significance was assessed using one-way ANOVA with Bonferroni's test for multiple comparisons. ***, *p* < 0.001; **, *p* < 0.01; §§, *p* < 0.01 *vs.* (*R*)-5-OH-DPAT; §, *p* < 0.05 *vs.* (*R*)-5-OH-DPAT; ###, *p* < 0.001 *vs. p*-tyramine; ##, *p* < 0.01 *vs. p*-tyramine. All data are shown as mean ± SEM.

To investigate how different ligand coupling profiles translate into proximate downstream signaling events, we used a G protein-coupled inward rectifier potassium (GIRK) channel activation assay in *Xenopus oocytes* co-expressing D_2_R, GIRK channels, RGS4, and when indicated, βarr2.^25,26^ GIRK channels are opened by βγ dimers, which are released upon G protein activation.^27^ Here, we measured agonist-induced increases in GIRK current as a readout of D_2_R-induced G protein activation. First, we investigated how the potencies of D_2_R agonists to evoke GIRK currents correlate with potency data obtained from BRET measurements. We found that the rank order of agonist potencies in the GIRK activation assay (Fig. 4A) overall agree with the order of potencies observed in the BRET-based G protein and βarr2 recruitment assays (Fig. 1A–D and 2A–D), *i.e.*; (*S*)-5-OH-DPAT ≥ dopamine > (*R*)-5-OH-DPAT > *p*-tyramine. We further tested the ability of βarr2 to desensitize the D_2_R-evoked G protein response, as measured at the level of GIRK channel activation, upon prolonged agonist exposure. Oocytes are known to express no detectable endogenous β-arrestins.^23^ In agreement, we observed that the GIRK channel responses to all four of the tested agonists decayed towards baseline at a faster rate in oocytes co-expressing exogenous βarr2, compared to dopamine-evoked responses in control oocytes which had not been injected with βarr2 cRNA (Fig. 4B and C). In the βarr2-expressing oocytes, the response decay rates observed with (*R*)-5-OH-DPAT and *p*-tyramine were slower than those with dopamine and (*S*)-5-OH-DPAT.

### A common mechanism for monoaminergic GPCRs

**Fig. 5:**
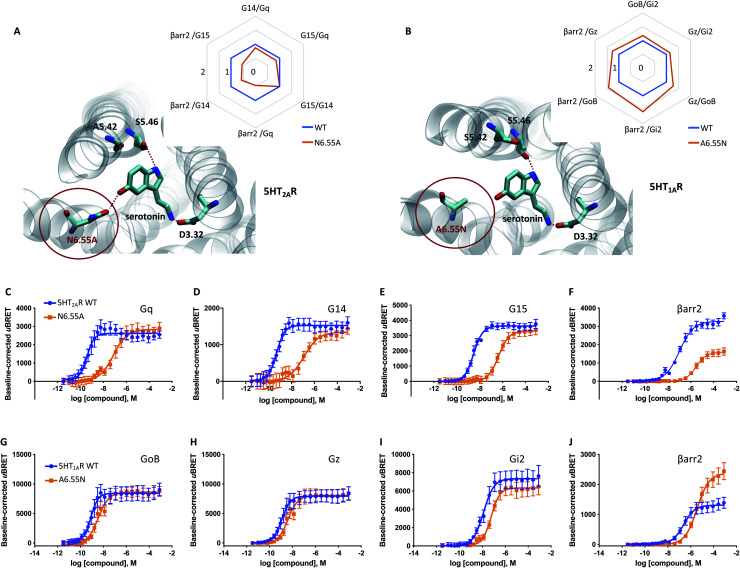
Relevance of H6.55 for neurotransmission at the 5-HT_1A_R and 5-HT_2A_R. (A) Binding mode and area under the curve (AUC)-based coupling ratios for the 5-HT_2A_R obtained from response curves in C to F. (B) Binding mode and AUC-based coupling ratio for the 5-HT_1A_R obtained from response curves in G to J. To eliminate observational bias linked to differences within biosensor assays (*e.g.* βarr2 *vs.* G_i_), we use the serotonin response at the WT receptor as internal standard for analyzing the AUC ratios. Note that obtained AUC ratios (*i.e.* the receptor's ability to couple to different effector proteins) is not affected by expression levels of the receptor (*i.e.* receptor concentration) which can change upon mutation. (C to F) Concentration–response curves of the serotonin-induced coupling of the 5-HT_2A_R to G_q_, G_14_, G_15_ and βarr2. (G to J) Concentration–response curves of the serotonin-induced coupling to G_oB_, G_z_, G_i2_ and βarr2. All concentration–response curves were generated from a minimum of 3 independent experiments. For corresponding pEC_50_ and *E*_max_ values see Table S3.†

Our data indicates that a ligand-mediated hydrogen bond network between TM5 and TM6 contributes to a balanced G protein and βarr2 coupling profile at the D_2_R. Within this contact network, specific interactions with TM6 seem to promote βarr2 recruitment. To investigate if this represents a common mechanism for other neurotransmitter receptors, we studied the action of serotonin on the serotonergic receptors 1A (5-HT_1A_R) and 2A (5-HT_2A_R). Similar to dopamine (Fig. 1N and O), serotonin is able to form simultaneous polar interactions with TM5 (position 5.46) and TM6 (position 6.55) in the 5-HT_2A_R^11^ (Fig. 5A). Importantly, we find that replacing the polar residue in position 6.55 by a structurally similar but non-polar residue (N6.55A) reduces the coupling potency to G proteins (G_q_, G_14_, G_15_) (Fig. 5C to E). However, for βarr2 we see that both coupling potency and efficacy are reduced (Fig. 5F). These concerted coupling alterations result in a preferential coupling of G proteins over βarr2 as reflected by the AUC-based coupling ratios (Fig. 5A). The highest coupling preference is observed for G_q_ over βarr2, which corresponds to a bias (10^ΔΔlog(*τ*/*K*_A_)^) of ∼10 fold based on the operational model (Table S4E†). This finding supports the notion that position 6.55 is a hotspot for modulating the coupling balance between βarr2 and G proteins. We further studied the significance of this position at the 5-HT_1A_R, which lacks a polar residue at position 6.55 (A6.55; see in Fig. 5B). According to our working model, introducing a polar residue should result in a higher βarr2 recruitment efficacy relative to the WT 5-HT_1A_R. In fact, our experiments show that an engineered A6.55N variant of the 5-HT_1A_R induces a slight reduction in coupling potency for all tested effector proteins (G_oB_, G_z_, G_i2_, βarr2) (Fig. 5G to J). However, we find a notable increase in coupling efficacy for βarr2 with unchanged efficacies for G protein coupling. Preferential coupling to βarr2 over G proteins for 5-HT_1A_R (A6.55N) is further illustrated *via* calculation of AUC-based coupling ratios (Fig. 5B). The highest coupling preference is observed for βarr2 over G_i2_, which corresponds to a 6 fold bias (10^ΔΔlog(*τ*/*K*_A_)^) based on the operational model (Table S4F†). Overall, our data suggest that amino acid variations at position 6.55 may serve as a way to fine-tune βarr recruitment to aminergic neurotransmitter receptors and, ultimately, regulate their desensitization, internalization and/or signaling.

## Discussion

By means of classical molecular dynamics simulation, enhanced sampling techniques, live-cell biosensor assays and the use of structurally related signaling probes, we have dissected the coupling profile of the neurotransmitter dopamine at the D_2_R. Using compounds such as *p*-tyramine, we find that exclusive TM5 interaction *via* the *p*-OH group (Fig. 1M) yields preferential G_oB_ coupling over βarr2 (Fig. 1D and R). Interestingly, a recent study by Sommer *et al.* identified hordenine – a compound found in beer – as a functionally biased D_2_R agonist.^28^ Hordenine, which differs from *p*-tyramine only by additional *N*,*N*-di-methyl groups, was shown to induce G_i_ protein activation (measured *via* cAMP assays) while antagonizing β-arrestin engagement downstream of D_2_R. These data are in line with our findings. As the authors measured the functional outcome as cAMP inhibition, no information is available about the specific G_i_-family member engaged. Using live-cell BRET-based biosensors, we tested the ability of hordenine to recruit different G_i_-family G protein subtypes as well as βarr2. We find a preferential G_oB_ coupling profile for hordenine, which seems to be related to exclusive TM5 interaction similar to *p*-tyramine (Fig. S6†).

We further conclude that D_2_R coupling to βarr2 in response to dopamine is attributed to additional contacts with position 6.55 in TM6 *via* its *m*-OH group (Fig. 1N and O). We demonstrate this by using rigid dopamine analogues ((*R*)/(*S*)-5-OH and (*R*)/(*S*)-7-OH-DPATs). Interestingly, depending on their chirality, enantiomeric DPATs are able to favor one binding mode with either simultaneous TM5/TM6 or exclusive TM5 interactions, resulting in a distinct coupling outcome. It is worth noting that the described mechanistic link between the binding mode and the coupling outcome for chiral DPATs may also explain recent findings for the signaling preference of extended ligands that include a chiral DPAT scaffold.^29^

A general overview of ligand-receptor contacts for dopamine-like and G protein biased compounds is provided by a contact heatmap (Fig. S5†). The heatmap highlights polar contacts (including water-mediated interactions) that are established at an energy minimum of ligand binding. As expected, we find strong interactions with key residues in TM3 (D3.32) and TM5 (S5.42 or S5.46) for studied ligands independently of their coupling properties. In contrast, polar interactions with H6.55 in TM6 are preferentially observed for ligands with an unbiased coupling profile for G proteins and βarr2 (dopamine-like). The contact heatmap reveals further polar contacts with TM7 (*i.e.* Y7.43) for dopamine, *p*- and *m*-tyramine but not for DPAT derivatives. As DPATs extend hydrophobic di-propyl groups towards TM7, they are not able to form such polar contacts. It is worth noting that our data suggests that polar contacts in TM7 are not important for coupling specificity as they are found in unbiased (*e.g.* dopamine) as well as G protein biased compounds (*e.g. p*-tyramine).

Electrophysiological experiments show that specific ligand coupling profiles propagate further downstream to the level of GIRK channel activation. An important observation is that compounds with G protein-coupling preference result in prolonged GIRK channel responses (Fig. 4 B and C). Mechanistically, these compounds likely stabilize a D_2_R conformational state with low affinity towards βarr2 and are therefore less susceptible to receptor desensitization which is reflected in prolonged GIRK openness. Ultimately, this finding provides additional evidence that (*R*)-5-OH-DPAT and *p*-tyramine exhibit a preference for G protein coupling over βarr2 compared to dopamine and (*S*)-5-OH-DPAT.

Interestingly, none of the tested compounds induce preferential coupling to βarr2. McCorvy *et al.* suggest that βarr2 bias in the D_2_R can be induced by exclusive contacts to Ile184 in the extracellular loop 2 (ECL2).^30^ Computing the frequency of Ile184 contacts for the ligands studied herein (*i.e.*, dopamine, *m*-/*p*-tyramine, DPATs and hordenine) indicates that these compounds are too small to establish significant contacts with this residue in the ECL2 (Table S6,† contact frequencies ≤ 15%). This would explain their preferential G protein coupling profile. Another study by Weichert *et al.* reports that βarr bias can be obtained by contacts in the extended binding pocket formed by TM2 and TM7.^31^ Again, this pocket is hardly within reach of the small molecular probes used in our study. However, these examples highlight the existence of multiple sites that modulate βarr2 coupling in the D_2_R.

Altogether, our data suggest that the mechanism underlying dopaminergic neurotransmission at the D_2_R involves a ligand-mediated polar network between TM5 and TM6. This translates into a physiological coupling response engaging effector proteins such as G_oB,_ G_i2_, G_z_, and βarr2. Noteworthily, we were able to support this notion by a mutational study of the key residue 6.55, which is part of this polar network (Fig. 3). This observation prompted us to investigate if the described structural features form part of a more general mechanism.

A sequence analysis of dopamine receptor subtypes (D_1_R to D_5_R, Fig. S7A†) reveals high conservation in key positions of the orthosteric binding site, such as serine residues in TM5 (S5.42 and S5.46) and a polar residue in TM6 (H6.55 or N6.55). It is tempting to speculate about potential coupling alterations induced by polar variations in position 6.55 comparing D_2,3,4_R (H6.55) *versus* D_1,5_R (N6.55). Interestingly, our mutational experiment carried out at the D_2_R and the obtained biased factor show that a H6.55N variation yields an unbiased coupling profile similar to the D_2_R WT (Table S4B†). This suggests that dopamine receptor subtypes D_2_,_3,4_R (H6.55) and D_1,5_R (N6.55) share a similar unbiased coupling profile (*i.e.* G proteins *vs.* βarr) between each other.

Most importantly, our data propose further that also serotonin-mediated neurotransmission at the 5-HT_2A_R is linked to simultaneous TM5/TM6 interaction (Fig. 5A). In this respect, we show that prevention of TM6 interaction by N6.55A mutation dampens βarr2 recruitment. Interestingly, this mechanism appears to be also conserved in adrenergic receptors such as the β_2_ adrenergic receptor (β_2_AR). In a recent study, authors focused on salmeterol, a β_2_AR-targeted drug used for the treatment of asthma and chronic obstructive pulmonary disease.^32^ They report that limited βarr recruitment of salmeterol is linked to reduced contacts with N6.55 in the β_2_AR which is in line with the proposed mechanism.

Curiously, not all aminergic GPCRs have a polar residue at position 6.55. A sequence alignment reveals that 44% of the receptors have a non-polar residue (retrieved from the GPCRdb^33^). According to our model, non-polar residues would partially dampen βarr2 coupling to the WT receptor. We indirectly prove this by introducing a polar residue into position 6.55 of the 5-HT_1A_R (A6.55N, Fig. 5B), which remarkably promotes βarr2 coupling over G proteins (Fig. 5G–J). This finding suggests that sequence diversity in position 6.55 (see Fig. S7†) may serve as an evolutionary mechanism to modulate βarr recruitment (and thus receptor internalization, desensitization and/or signaling) for different neurotransmitter receptors.

Beyond a general implication of H6.55 in βarr2 recruitment, we find that H6.55 may also have relevance for the coupling of G protein subtypes to the D_2_R. Relative to dopamine, compounds lacking contact to H6.55 (*i.e.*, *p*-tyramine, hordenine, (*R*)-5-OH-DPAT or (*S*)-7-OH-DPAT) appear to provoke a greater reduction in D_2_R coupling to G_i2_ and G_z_*vs.* G_oB_ (Fig. 1, 2 and S6†). Our data are in line with a previous mutational study by Tschammer *et al.* in which authors report a link between H6.55 interaction and G protein coupling specificity.^21^ Interestingly, this link seems to be also conserved in the adrenergic receptors. For instance, mutation of position 6.55 in the α_2A_ adrenergic receptor (α2AAR) leads to a switch of its dual coupling profile (G_s_ and G_i_) to a preferential G_s_ coupling.^34^ Note that this tendency does not appear to be strongly conserved in the 5-HT_1A_R since although a A6.55N mutation enhanced βarr2 recruitment, we find only a marginal impact on G protein coupling across G_oB_, G_z_ and G_i2_ proteins (Fig. 5G–J).

## Conclusion

In this study, we use all-atom molecular simulation combined with live-cell biosensor and electrophysiological assays to dissect the molecular mechanism of dopaminergic neurotransmission at the prototypical neurotransmitter receptor D_2_R. Our work identifies specific structural determinants of neurotransmission that translate into the activation of specific D_2_R effectors (*i.e.*: G_oB,_ G_i2_, G_z_ and βarr2). Most importantly, our study indicates that the identified molecular features govern the coupling outcome of serotonin as well as adrenergic receptors and are likely shared by other aminergic neurotransmitter receptors. Furthermore, our work highlights how nature deploys sequence variations to fine-tune the coupling outcome of neurotransmitter receptors across different receptor subtypes. Ultimately, obtained structural insights provide novel hints for the rational design of more efficient and safer drugs for this important drug target class.

## Method

### Homology modelling of the active state of D2R

The canonical sequence of the D_2_ receptor was obtained from the Uniprot database (accession number: P14416). The sequence was aligned with that of the β_2_AR obtained from the template structure (PDB code: 3P0G) using Clustal Omega.^35^ The alignment was manually refined to maintain the position of highly conserved residues. The first 36 residues of the D_2_R sequence were truncated as they formed a flexible N-terminal tail. The long intracellular loop 3 was shortened and the ends were fused. Based on the obtained alignment, we generated 500 models using homology model tool implemented in the MOE package (http://www.chemcomp.com). The best model was selected based on the lowest DOPE (Discrete Optimized Protein Energy) score. The hydrogen network was optimized at pH 7 using Protonate3D^36^ available in the MOE package (http://www.chemcomp.com). It is worth noting that the active D_2_R in complex with G_i_ (PDB code: 6VMS) has been recently obtained while this manuscript was in preparation.^16^ This structure provides high-resolution insights into the orthostatic binding pocket of the active D_2_R. Importantly, the backbone atoms for residues in the binding pocket that complex dopamine adopt a RMSD value of only 0.895 Å which supports the high quality of our computational receptor model (Fig. S1†).

### Generation of protein–ligand complexes

The starting poses of dopamine were obtained by docking with GOLD software.^37^ The atoms of the protein were kept rigid, while the ligand was allowed flexibility. A positional restraint was included, to take into account only poses in which the ligand forms polar interactions with D3.32. Using this protocol 900 poses were generated per ligand. The poses were scored with goldscore, and rescored using the plp score. Afterwards the best poses for each ligand were picked taking into account the scoring, as well as visual inspection. Each ligand–protein complex was optimized during a molecular dynamics run in conditions of constant pressure (see below for a description). Then, initial poses for *m*-tyramine and *p*-tyramine were obtained by removing the *meta* or *para* hydroxyl group of the dopamine pose obtained after the molecular dynamics optimization.

Structural models for the 5-HT_1A_R and 5-HT_2A_R in complex with serotonin were obtained based on the work from Martí-Solano *et al.*^11^ The structures were subjected to a short minimization using the MOE package (http://www.chemcomp.com).

### Molecular dynamics simulations

To generate starting systems, ligands (in accordance with their poses obtained in the previous step) were placed in the active state model of the D_2_R. To ensure proper orientation of the receptor in the membrane, the complexes were aligned to the structure used as the template (PDB code: 3P0G) obtained from the OPM database.^38^ Subsequently, we used the output aligned structures to generate systems for molecular dynamics. The systems were generated using CHARMM-GUI.^39^ The receptor was embedded in a ∼80 × 80 Å POPC bilayer. The resulting complex was solvated with ∼8200 TIP3 molecules. The ionic strength of the solution was kept at 0.15 M NaCl. Additional chloride ions were added in order to keep the charge of the system neutral. Disulfide bonds were introduced in accordance with data obtained from the Uniprot database. Parameters for the simulation were obtained from the CHARMM36 forcefield.^40^ Parameters for the ligand were assigned from the CGenFF forcefield automatically by the ParamChem tool implemented in CHARMM-GUI.^41,42^ The systems were first equilibrated in conditions of constant pressure (NPT, 1.01325 bar) for 100 ns. Over the first half of the simulation we applied constraints to the backbone atoms. The constraints were gradually released over the first 50 ns of the simulation. After the NPT step, we have carried out simulations in conditions of constant volume (NVT) of the system for 600 ns in 4 replicates. The simulations were run in ACEMD.^43^ We used a time-step of 4 fs. Such a large time-step was possible due to the hydrogen mass repartitioning scheme being employed in ACEMD.^44^ A non-bonded interaction cutoff was set at 9 Å. A smooth switching function for the cut-off was applied, starting at 7.5 Å. Long-distance electrostatic forces were calculated using the Particle Mesh Ewald algorithm. The algorithm had grid spacing of 1 Å. The bond lengths of hydrogen atoms were kept constrained using the RATTLE algorithm. Simulations were carried out at a temperature of 300 K in periodic boundary conditions. A summary of molecular dynamics simulations carried out in this study is found in Table S1.†

### Metadynamics

Metadynamics is a biased dynamics technique widely used to improve sampling for free energy calculations over a set of multidimensional reaction coordinates which would not be sampled exhaustively with normal unbiased simulations.^45^ It is implemented in the molecular dynamics software ACEMD using the PLUMED plugin interface.^46^ Here, we use this approach to construct the complete energetic binding landscape of dopamine and its derivatives. For this, we used as collective variables the distance of oxygen atoms of *m*- and/or *p*-OH groups of the ligand to the OG atom of S5.42 (CV1) and/or S5.46 (CV2) in TM5 (Fig. S2†). The metadynamics parameters were set to a Gaussian hill height of 0.1 kcal mol^−1^ with a spread of 0.1 Å for the CV1 and/or CV2. The deposition rate was one hill every 4 ps and a well-tempered bias factor of 10. To ensure exhaustive sampling within the orthosteric binding site, we set a restraining potential with an energy constant kappa of 100 that starts acting when the distance of the CV1 or CV2 exceeds 12 Å. In addition, we used the multiple walker approach,^47^ in which 6 walkers simultaneously explore the same free-energy landscape and interact by contributing to the same history-dependent bias potential every 20 ps. Walkers for each ligand were obtained from unbiased NVT simulations (protocol described in the previous step). Each system was simulated for an accumulated time of at least 1.1 μs or until the free-energy landscape converged. General simulation parameters were kept as described for the production run in the previous section. Ultimately, we plotted the energies as a function of the distance between the ligand's oxygen group and S5.42 or S5.46 (Fig. 1 and 2). In order to ensure the convergence of our metadynamics simulation, we monitored the changes of the free energy profile along the simulation time. For this, we computed the free energy every 20 000 deposited Gaussian yielding 15 to 20 graphs per simulation setup.

### Experimental validation using a cell-based assay

Bioluminescence resonance energy transfer (BRET)-based biosensor assays (bioSensAll™) were conducted at Domain Therapeutics NA Inc. (Montreal, QC, Canada). Assays were performed in HEK-293T cells, which were cultured in Dulbecco's Modified Eagle Medium (DMEM) (Wisent # 319-015-CL) supplemented with 1% penicillin-streptomycin G (Wisent; cat# 450-201-EL) and 10% fetal bovine serum (Wisent # 090150) and maintained at 37 °C with 5% CO_2_. All biosensor-coding plasmids and related information are the property of Domain Therapeutics NA Inc: GAPL-G_i2_ (cat# DTNA A29), GAPL-, GAPL-G_oB_ (cat# DTNA A32), GAPL-G_z_ (cat# DTNA A33) and βarr2-PM + GRK2 WT (cat# DTNA A46). Information pertaining to the βarr2-PM biosensor has been previously published.^48^ Experiments with the D_2_R were performed with the long isoform (canonical sequence, post-synaptic localization; Uniprot P14416-1). All receptor point mutations were produced by TOP Gene Technologies Inc. (Montreal, QC, Canada). Transfections were performed using 25 kDa linear PEI (Polysciences, Warrington, PA) at a 3 : 1 μl of PEI/μg of DNA ratio. Briefly, DNA and PEI were diluted separately in 150 mM NaCl, mixed and then incubated for at least 20 minutes at room temperature (note: total amount of DNA transfected was adjusted to a final quantity of 2 μg with salmon sperm DNA (Invitrogen)). During the 20 minute incubation, HEK-293T cells were detached, counted and re-suspended into cell culture medium to a final density of 350 000 cells per mL. At the end of the 20 minute incubation, DNA/PEI complexes were added to cells followed by a gentle mixing. Cells were subsequently distributed in cell culture-treated 96-well plates (White Opaque 96-well Microplates, Greiner, cat# 655) at a density of 35 000 cells per well (*i.e.*, 100 μl of cell suspension per well) and incubated at 37 °C for 48 h. At 48 hours post-transfection, the transfection medium was removed and cells were washed once with 100 μl of Tyrode–Hepes buffer (Sigma, cat# T2145 + H9136) per well. Wash buffer was then replaced by 100 μl of fresh Tyrode–Hepes buffer per well and plates were incubated for 60 min at room temperature. At the end of this equilibration period, 10 μl of 20 μM e-Coelenterazine Prolume Purple (Methoxy e-CTZ; Nanolight, # 369) was added to each well followed immediately by the addition of increasing test compound concentrations. Cells were then incubated at room temperature for 10 minutes and BRET readings subsequently collected with a 0.4 s integration time on a Synergy NEO plate reader (BioTek Instruments, Inc., USA; filters: 400 nm/70 nm, 515 nm/20 nm). The BRET signal was calculated as the ratio of GFP10 emission to RLucII emission. All resulting dose response curves are represented as baseline-corrected *u*BRET (*i.e.*, baseline *u*BRET values subtracted from curves).

### Calculation of the AUCs and their pathway ratios (AUC-based coupling ratios)

As an approximation of pathway-specific receptor coupling preferences, we use the area under the curve (AUC) of the concentration–response curves. This AUC takes into account potency (EC_50_), efficacy (*E*_max_) and the Hill slope. AUCs were obtained using GraphPad Prism 6 software. In order to facilitate the comparison of individual pathways for tested compounds, we computed the AUC-based coupling ratios for all combinations of individual effector proteins (*e.g.* AUC_βarr2_*vs.* AUC_Gz_, AUC_βarr2_*vs.* AUC_GoB_*etc.*). For D_2_R, coupling ratios for dopamine are used as the reference to which all other dopaminergic ligand coupling ratios are normalized. For 5-HT mutant receptors, the coupling ratios for the WT receptor in response to serotonin are used as reference (Fig. 5A and B). Further details on computation and raw data are found in the supplemental material (Table S7 and Fig. S8†).

### Purchased compounds

(*R*)-7-OH-DPAT: Cedarlane (Axon Medchem # 1013), (*S*)-7-OH-DPAT: Cedarlane (Axon Medchem # 1014), (*R*)-5-OH-DPAT: Cedarlane (Axon Medchem # 1007), (*S*)-5-OH-DPAT: Cedarlane (Axon Medchem # 1008), hordenine: Sigma # 04476, *m*-tyramine hydrochloride: Sigma # D017, Lot: 063K4620, *p*-tyramine hydrochloride: Sigma # T90344, noradrenaline hydrochloride: Sigma # 74480, serotonin hydrochloride: Sigma # H9523, dopamine hydrochloride: Sigma # H8502.

### Operational model of bias

The operational model of agonism was used to estimate ligand bias when applicable according to recently published protocols.^23,24^ All data were analyzed using the nonlinear curve fitting functions in GraphPad Prism (v6.0; GraphPad Software, La Jolla, CA). Ligand bias was quantified by analyzing the concentration–response curves using the operational model of agonism according to the equation
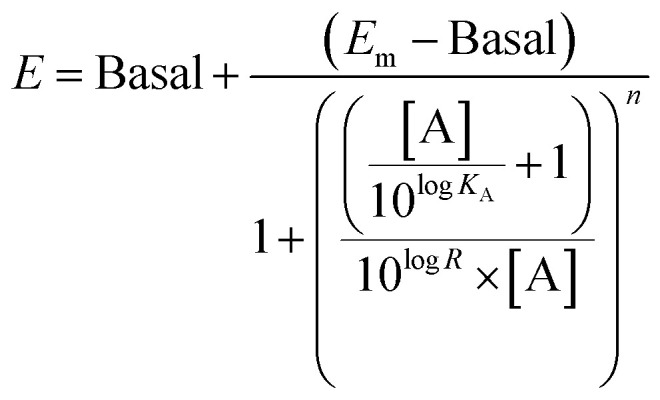
where: *E* = effect of the ligand; [A] = concentration of agonist; *E*_m_ = maximal possible response of the system; Basal = basal level of response in the absence of agonist; log *K*_A_ = logarithm of the functional equilibrium dissociation constant of the agonist; *n* = slope of the transducer function that links occupancy to response; log *R* = logarithm of the transduction ratio, *τ*/*K*_A_, where *τ* is an index of the coupling efficiency (or efficacy) of the agonist.

The following parameters were used for fitting of all families of agonist curves at each pathway to the model: Basal, *E*_m_, and *n* were shared between all agonists; for full agonists, log *K*_A_ was constrained to a value of zero; for partial agonists, log *K*_A_ was directly estimated by the curve fitting procedure. The log *R* [*i.e.*, log(*τ*/*K*_A_)] parameter was estimated as a unique measure of activity for each agonist.

The logarithmic form of the transduction ratios (*τ*/*K*_A_) was then obtained from fitted concentration–response curves for the recruitment of G_oB_ and βarr2 (determined BRET-based assay). To account for cell-system-dependent factors between different assay systems, the transduction coefficients (τ/*K*_A_) were normalized to the response of the reference agonist dopamine:
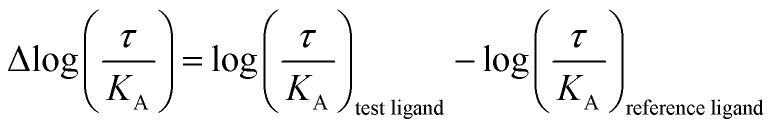
Coupling bias was obtained by calculating the difference between two investigated pathways for the same ligand:
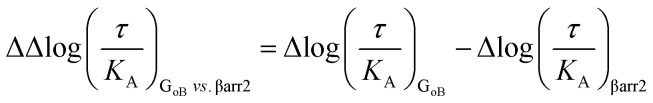

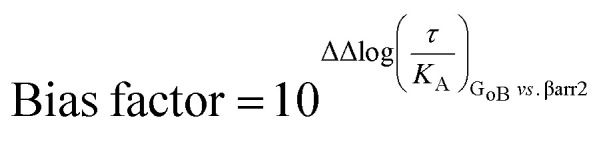


### Molecular biology for electrophysiology experiments

Human GIRK1 (Kir3.1) and GIRK4 (Kir3.4) cDNA (provided by Dr Terence Hebert, McGill University, Montreal, Canada) and RGS4 (from the cDNA Resource Center, Bloomsberg, PA; https://www.cdna.org) were in pcDNA3.1+. cDNA encoding the human dopamine D_2S_ receptor and βarr2 (ARRB2; synthesized by Genscript, Piscataway, NJ) were in pXOOM (a gift from Dr Søren-Peter Olesen, University of Copenhagen, Denmark). For *in vitro* transcription, plasmids were linearized with the appropriate restriction enzymes (GIRK 1/4, NotI; RGS4, D_2S_ and βarr2, XhoI) and transcribed *in vitro* using the T7 mMessage mMachine kit (Ambion, Austin, TX). cRNA concentration and purity were determined using a spectrophotometer.

### Oocyte isolation and injection

Oocytes were surgically isolated from female African clawed toads, *Xenopus laevis*, or purchased from EcoCyte Bioscience (Castrop-Rauxel, Germany), and injected with cRNA as previously described.^24^ The surgical procedures had been approved by the Swedish National Board for Laboratory Animals. 1 ng of each GIRK1/4 subunit cRNA, 40 ng of RGS4 cRNA, 0.2 ng of dopamine D_2S_ receptor cRNA, and when used 5.6 ng of βarr2 cRNA, were injected per oocyte. RGS proteins are GTPase-activating proteins expressed in native tissues, which speed up the G protein cycle such that GIRK channel activity more closely follows receptor occupancy by agonist.

### Electrophysiology

Following oocyte injection with cRNA and 6 days of incubation at 12 °C, electrophysiology experiments were conducted using the parallel two-electrode voltage-clamp apparatus, OpusXpress 6000A (Molecular Devices, San José, CA). Continuous perfusion, mediated by Minipuls 3 peristaltic pumps (Gilson, WI), was maintained at 0.5 ml min^−1^ (for concentration–response experiments) or 3.5 ml min^−1^ (for desensitization experiments). Data were acquired at membrane potentials of −80 mV and sampled at 156 Hz using OpusXpress 1.10.42 software (Molecular Devices). To increase the inward rectifier potassium channel current at negative potentials, a high-potassium extracellular buffer was used (in mM: 64 NaCl, 25 KCl, 0.8 MgCl_2_, 0.4 CaCl_2_, 15 HEPES, 1 ascorbic acid, adjusted to pH 7.4), yielding a potassium reversal potential of about −40 mV. Ascorbic acid prevented the spontaneous oxidation of dopamine, which was purchased from Sigma-Aldrich (St. Louis, MO).

### Data analysis

Electrophysiological data were analyzed in Clampfit 10.6 (Molecular Devices). Concentration–response curves were calculated using the variable-slope sigmoidal functions in GraphPad Prism 6. In each cell, the current responses evoked by each concentration of agonist were normalized to the mean response evoked by 1 μM dopamine in oocytes from the same batch (*i.e.*; same toad and preparation date). Normalized concentration–response data were fitted to the following equation:
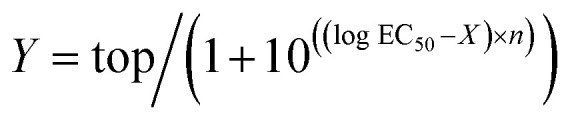
where *Y* is the normalized GIRK current response, top is the maximal response to the agonist in question, *X* is the logarithm of agonist concentration, and *n* is the Hill slope.

Differences in response decay rates between agonists and conditions were evaluated for statistical significance in GraphPad Prism 6 using one-way ANOVA with Bonferroni's test for multiple comparisons.

## Data availability

All data needed to evaluate the conclusions are present in the paper, the supplementary material or have been made available at GPCRmd^49^ (https://submission.gpcrmd.org/dynadb/publications/1474/).

## Author contributions

TMS, JS performed computational experiments which guided wet lab experiments carried out by AM. TMS, AM, BB and JS analysed and interpreted obtained results. MTF carried out the sequence analysis of receptors and their natural genetic variants. RA carried the GIRK channel experiments under supervision from KS. TMS, MTF and JS prepared figures for publication. TMS and JS wrote the manuscript with input from AM, KS and BB. JS supervised and coordinated the project.

## Conflicts of interest

The authors declare no conflict of interest.

## Supplementary Material

SC-012-D1SC00749A-s001
