# Supplementary material for: Mechanistic insights into dopaminergic and serotonergic neurotransmission – concerted interactions with helices 5 and 6 drive the functional outcome
Source: Chem Sci. 2021 Jul 2;12(33):10990–1003. doi: 10.1039/d1sc00749a (PMC8386650; doi:10.1039/d1sc00749a)
Supplement: SC-012-D1SC00749A-s001 [file SC-012-D1SC00749A-s001.pdf]

Electronic Supplementary Material (ESI) for Chemical Science.
This journal is © The Royal Society of Chemistry 2021

Supporting Information

Mechanistic insights into dopaminergic and serotonergic neurotransmission - concerted interactions with helices 5 and 6 drive the functional outcome

Tomasz Maciej Stepniewski[1,2#], Arturo Mancini[3#*], Richard Ågren[4], Mariona Torrens-Fontanals[1], Meriem Semache[3], Michel Bouvier[5,6], Kristoffer Sahlholm[4,7], Billy Breton[3,6] and Jana Selent[1*]

[1]Research Programme on Biomedical Informatics (GRIB), Hospital del Mar Medical Research Institute (IMIM) - Pompeu Fabra University (UPF), Dr. Aiguader 88, E-08003, Barcelona, Spain
[2]InterAx Biotech AG, PARK innovAARE, 5234 Villigen, Switzerland
[3]Domain Therapeutics NA Inc. 7171 Frederick-Banting, Local 3209, Saint-Laurent (QC) H4S 1Z9, Canada
[4]Department of Neuroscience, Karolinska Institute, Stockholm, Sweden
[5]Department of Biochemistry and Molecular Medicine, Université de Montréal, Montreal, QC H3C 3J7, Canada
[6]Institute for Research in Immunology and Cancer (IRIC), Université de Montréal; Montréal, Québec, H3T 1J4, Canada
[7]Department of Integrative Medical Biology, Wallenberg Centre for Molecular Medicine, Umeå University, 901 87, Umeå, Sweden

[#]Both authors contributed equally to this work.
*Corresponding authors:
e-mail: jana.selent@upf.edu
e-mail: amancini@domaintherapeutics.com

Table of Contents

1. Summary of molecular dynamics simulations

2. General binding mode of dopamine by unbiased molecular dynamics simulations

3. Site directed mutagenesis data

4. Metadynamics and applied bias

5. List of pEC50 and Emax

6. Operational model of bias

7. Conformational variability of ligand binding

8. Chirality-driven binding mode of (R)- and (S)- DPATs

9. Ligand interactions with the extracellular loop 2 (ECL2)

10. Contact heatmaps of ligand binding at energetic minimum

11. Binding signature of hordenine and its coupling outcome

12. Sequence analysis across dopamine, serotonin and adrenergic receptors

13. Experimental Procedures

1. Summary of molecular dynamics simulations

Table S1. Molecular dynamics simulation: classical unbiased simulation and metadynamics enhanced sampling

Compound	Comment	Simulation time [µs]
dopamine	unbiased simulation<br><br>protonation state 1: HSD6.55<br>protonation state 2: HSE6.55	 <br>4 x 0.6<br>4 x 0.6
p-tyramine	unbiased simulation	4 x 0.6
m-tyramine	unbiased simulation	4 x 0.6
(R)-7-OH-DPAT	unbiased simulation	4 x 0.6
(S)-7-OH-DPAT	unbiased simulation	4 x 0.6
(R)-5-OH-DPAT	unbiased simulation	4 x 0.6
(S)-5-OH-DPAT	unbiased simulation	4 x 0.6
dopamine	metadynamics<br>bias on m-OH	3.04
dopamine	metadynamics<br>bias on p-OH	1.45
p-tyramine	metadynamics<br>bias on p-OH	2.40
m-tyramine	metadynamics<br>bias on m-OH	1.25
(R)-7-OH-DPAT	metadynamics<br>bias on 7-OH	1.50
(S)-7-OH-DPAT	metadynamics<br>bias on 7-OH	1.35
(R)-5-OH-DPAT	metadynamics<br>bias on 5-OH	1.30
(S)-5-OH-DPAT	metadynamics<br>bias on 5-OH	3.20
hordenine	metadynamics<br>bias on p-OH	3.58
	**Total simulation time**	**38.27**

2. General binding mode of dopamine by unbiased molecular dynamics simulations

A first approximation of the dopamine binding mode was obtained by unbiased molecular dynamics simulation (Figure S1). The initial pose of dopamine, was obtained using the structure of adrenaline bound to the active β2-adrenergic receptor (PDB code: 4LDO). The binding of dopamine is mediated via a salt bridge formed between the protonated nitrogen of dopamine and the highly conserved D3.32. In addition, meta and para hydroxyl groups establish polar contacts with TM5 and TM6. In particular, polar interactions with TM5 are in agreement with site directed mutagenesis[1–5] and computational[6] studies. Note that interactions with TM6 are mediated by a water molecule at times.

Previous site directed mutagenesis studies[7] propose that H6.55 is important in binding several D_2R ligands. At the simulated pH 7.0, H6.55 can exist in two transient protonation states. Individual protonation states are driven by its environment such as a completely solvated pocket or a pocket occupied by diverse ligands. We have carried out parallel simulations of the dopamine-D_2R complex, to compare how the protonation state impacts dopamine binding. Thereby, HSD corresponds to the histidine with protonation on δ1 whereas HSE corresponds to the histidine with protonation on ε2. The results show that in the HSD state this residue establishes more frequently polar interactions with dopamine. Thus H6.55 was assigned the HSD protonation state in all of the subsequent simulations.

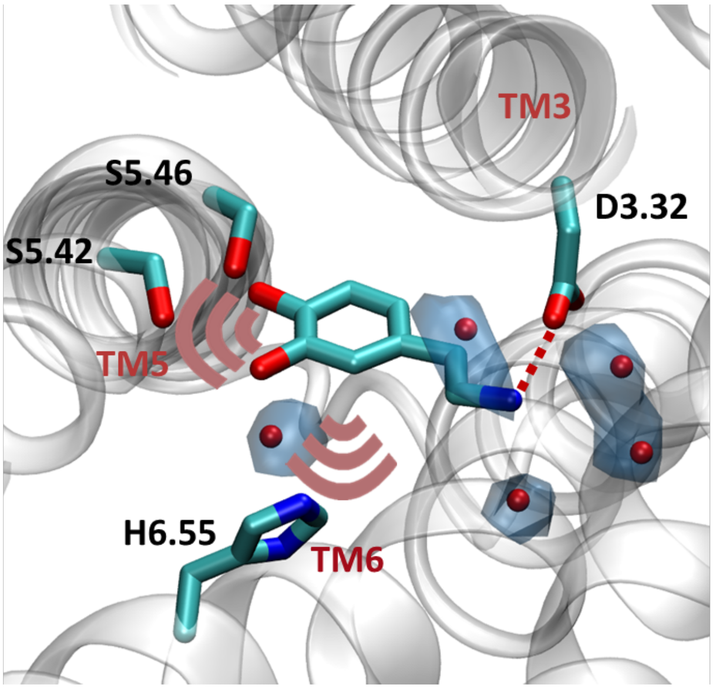

Figure S1. General binding mode of dopamine. Red dashed lines: salt bridge, red radar: polar contacts, blue surface: occupancy map of water is calculated over classical unbiased simulation using the volmap tool implemented in VMD software package.[8]

3. Site directed mutagenesis data

During the last decades, several mutagenesis studies have explored the impact of polar residues (S5.42, S5.43 and S5.46) in TM5 for binding of dopamine analogues. While they agree in TM5 as an important anchor point for binding of dopamine analogues, deviating results are obtained for individual residues. Here we provide a selection of references in Table S2. It is worth noting that mutation-induced alteration of ligand binding (Table S2A) does not always correlate to an altered ligand efficacy even in the same experimental setup (summarized in Table S2B). For instance, a S5.46A mutation reduces only slightly dopamine binding while almost abolishing ligand efficacy.[2] One possible explanation is that the S5.46A mutation affects signaling in a manner independent of ligand-receptor contacts (i.e. by disturbing a conserved ionic lock within the receptor). In addition, the slight effect on the binding affinity can be induced by water-mediated indirect interaction or by a general perturbation of the hydrogen bonding network around the ligand.

Table S2A. Impact of mutations of serine residues in TM5 on binding of dopamine and its analogues at the D_2R. The impact of a mutation is reported as the fold change between the WT and MT. A decrease is denoted with an arrow facing downwards (↓).

Single mutants	Isoform	Assay type	S5.42A	S5.46A	reference
Dopamine	long	K_i versus [^{3}H]spiperone	77↓	2↓	[1]
Dopamine	long	$K_{0,5}$ versus [^{3}H]N-methylspiperone	177↓	8↓	[2]
Dopamine (low affinity state)	short	K_L versus [^{3}H]spiperone	250↓	4↓	[4]
Dopamine (high affinity state)	short	K_H versus [^{3}H]spiperone	400↓	4↓	[4]
Dopamine	short	K_i versus ['251]epidepride	48↓	2.6↓	[5]
p-tyramine	short	K_i versus ['251]epidepride	2↓	insignificant	[5]
m-tyramine	short	K_i versus ['251]epidepride	insignificant	insignificant	[5]

Table S2B. Impact of mutations of serine residues in TM5 on the signaling response elicited by dopamine and its analogues at the D_2R**.** The impact of a mutation is reported as the fold change between the WT and MT. A decrease is denoted with an arrow facing downwards (↓) and an increase with an arrow facing upwards (↑).

Single mutants	Isoform	Assay type	S5.42A	S5.46A	reference
Dopamine	short	Inhibition of isoproterenol-stimulated cAMP accumulation [EC50]	220↓	18↓	[5]
Dopamine	short	Inhibition of isoproterenol-stimulated cAMP accumulation [EC50]	667↓	1,6↓	[4]
Dopamine	long	Inhibition of forskolin-stimulated cAMP accumulation [EC50]	> 1000↓ (no observable inhibition)	> 1000↓ (no observable inhibition)	[2]
Dopamine	long	Potency in affecting GTPγS binding [EC50]	6,29↓	no observable binding	[2]
Dopamine	short	Inhibition of isoproterenol-stimulated cAMP accumulation [Emax]	1,12↓	1,02↑	[5]
Dopamine	short	Inhibition of isoproterenol-stimulated cAMP accumulation [Emax]	1,03↓	1,16↑	[4]
Dopamine	long	Inhibition of forskolin-stimulated Cox accumulation	20↓	5↓	[2]
Dopamine	short	Ligand-stimulated binding of [^{35}S]GTPγS	1,03↓	1,25↓	[4]
p-tyramine	short	Inhibition of isoproterenol-stimulated cAMP accumulation [EC50]	no significant change in respect to WT	no significant change in respect to WT	[5]
p-tyramine	short	Inhibition of isoproterenol-stimulated cAMP accumulation [Emax]	no significant change in respect to WT	no significant change in respect to WT	[5]
m-tyramine	short	Inhibition of isoproterenol-stimulated cAMP accumulation [EC50]	no significant change in respect to WT	no significant change in respect to WT	[5]
m-tyramine	short	Inhibition of isoproterenol-stimulated cAMP accumulation [Emax]	no significant change in respect to WT	no significant change in respect to WT	[5]

4. Metadynamics and applied bias

To exhaustively sample all potential binding modes, we biased contacts of m- and/or p-OH groups to polar residues in TM5 (S5.42 and S5.46) with a distance restraint of 12 Å. In addition, we applied a constraint to maintain the protonated nitrogen within a distance of 5 Å of the carboxylic group the D3.32. In initial experiments, we also biased the distance of aromatic substituents to S5.43. However, we did not obtain any binding peaks, therefore neglecting this interaction for following experiments.

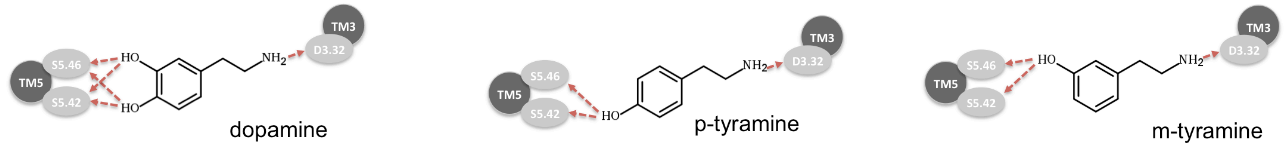

Figure S2. Metadynamics and applied bias.

5. List of pEC50 and Emax

Table S3A. Ligand potencies (pEC50) and corresponding standard errors (SE) for different coupling partners.

	G_{oB}		G_z		G_{i2}		βarr2	
**compound**	pEC50	SE	pEC50	SE	pEC50	SE	pEC50	SE
dopamine	9.35	0.04	8.51	0.04	8.30	0.05	7.33	0.07
m-tyramine	7.29	0.12	6.31	0.05	6.22	0.02	5.46	0.20
p-tyramine	5.00	0.03	4.20	0.10	4.23	0.14	N/D	N/D
hordenine	5.08	0.02	4.67	0.34	4.50	0.20	N/D	N/D
(R)-5-OH-DPAT	8.64	0.03	7.84	0.06	7.73	0.07	6.92	0.22
(S)-5-OH-DPAT	10.63	0.06	9.62	0.07	9.51	0.05	8.57	0.08
(R)-7-OH-DPAT	9.97	0.07	9.15	0.07	8.74	0.06	8.06	0.07
(S)-7-OH-DPAT	8.26	0.05	7.08	0.07	6.90	0.04	5.79	0.20

Table S3B. Ligand efficacies (Emax) and corresponding standard errors (SE) for different coupling partners.

	G_{oB}		G_z		G_{i2}		βarr2	
**compound**	Emax	SE	Emax	SE	Emax	SE	Emax	SE
dopamine	100.00	0.83	100.00	0.93	100.00	1.27	100.00	1.94
m-tyramine	98.79	3.22	92.11	1.83	83.27	0.80	47.98	4.18
p-tyramine	90.98	1.71	61.43	5.10	47.00	5.61	N/D	N/D
hordenine	79.74	1.11	26.82	4.86	18.33	2.59	N/D	N/D
(R)-5-OH-DPAT	104.30	0.82	86.60	2.87	72.27	1.46	41.63	2.69
(S)-5-OH-DPAT	108.40	1.12	100.80	1.32	99.38	1.04	96.31	1.71
(R)-7-OH-DPAT	107.50	1.39	97.44	1.30	97.36	1.40	85.38	1.60
(S)-7-OH-DPAT	106.30	1.40	97.05	2.21	94.56	1.43	77.27	6.43

Table S3C. Ligand efficacies (Emax) and corresponding standard errors (SE) for dopamine.

	G_{oB}		βarr2	
**compound**	Emax	SE	Emax	SE
WT D_2R	100.00	1.61	100.00	2.23
H393F D_2R	90.57	1.78	33.55	4.49
H393N D_2R	109.10	1.46	108.30	5.49

Table S3D. Ligand potency (pEC50) and corresponding standard errors (SE) for dopamine.

	G_{oB}		βarr2	
**compound**	pEC50	SE	pEC50	SE
WT D_2R	9.12	0.06	7.35	0.07
H393F D_2R	6.86	0.05	5.74	0.29
H393N D_2R	7.62	1.72	5.67	0.12

Table S3E. Ligand efficacies (Emax) and corresponding standard errors (SE) for (R)-7-OH-DPAT.

	G_{oB}		βarr2	
**compound**	Emax	SE	Emax	SE
WT D_2R	100.00	0.82	100.00	1.77
H393F D_2R	97.55	0.87	46.21	2.78
H393N D_2R	109.70	1.06	107.60	2.03

Table S3F. Ligand potency (pEC50) and corresponding standard errors (SE) for (R)-7-OH-DPAT.

	G_{oB}		βarr2	
**compound**	pEC50	SE	pEC50	SE
WT D_2R	9.97	0.04	8.07	0.06
H393F D_2R	9.02	0.04	7.57	0.18
H393N D_2R	9.55	0.04	7.61	0.06

Table S3G. Ligand efficacies (Emax) and corresponding standard errors (SE) for (S)-5-OH-DPAT.

	G_{oB}		βarr2	
**compound**	Emax	SE	Emax	SE
WT D_2R	100.00	0.70	100.00	1.51
H393F D_2R	97.33	1.17	41.29	1.15
H393N D_2R	111.70	0.98	131.20	2.21

Table S3H. Ligand potency (pEC50) and corresponding standard errors (SE) for (S)-5-OH-DPAT.

	**G_{oB}**		**βarr2**	
**compound**	pEC50	SE	pEC50	SE
WT D_2R	10.77	0.04	8.66	0.06
H393F D_2R	10.15	0.07	8.81	0.09
H393N D_2R	10.84	0.05	8.74	0.06

Table S3I. Ligand efficacies (Emax) and corresponding standard errors (SE) for the 5-$HT_{2A}R$

	**G_q**		**G_{14}**		**G_{15}**		**βarr2**	
**compound**	Emax	SE	Emax	SE	Emax	SE	Emax	SE
5-$HT_{2A}R$ WT	100.00	2.05	100.00	1.07	100.00	0.98	100.00	1.69
5-$HT_{2A}R$ H343A	108.80	2.26	88.02	2.40	90.95	0.64	48.71	0.35

Table S3J. Ligand potency (pEC50) and corresponding standard errors (SE) for the 5-$HT_{2A}R$

	**G_q**		**G_{14}**		**G_{15}**		**βarr2**	
**compound**	pEC50	SE	pEC50	SE	pEC50	SE	pEC50	SE
5-$HT_{2A}R$ WT	9.43	0.06	9.30	0.03	8.53	0.03	6.99	0.06
5-$HT_{2A}R$ H343A	7.13	0.07	6.92	0.09	6.35	0.02	5.52	0.02

Table S3K. Ligand efficacy (Emax) and corresponding standard errors (SE) for the 5-$HT_{1A}R$

	**G_{oB}**		**G_z**		**G_{i2}**		**βarr2**	
**compound**	Emax	SE	Emax	SE	Emax	SE	Emax	SE
5-$HT_{1A}R$ WT	100.00	0.65	100.00	1.09	100.00	0.92	100.00	1.26
5-$HT_{1A}R$ A365N	101.40	1.97	102.50	1.89	86.17	0.92	182.10	2.27

Table S3L. Ligand potency (pEC50) and corresponding standard errors (SE) for the 5-$HT_{1A}R$

	**G_{oB}**		**G_z**		**G_{i2}**		**βarr2**	
**compound**	pEC50	SE	pEC50	SE	pEC50	SE	pEC50	SE
5-$HT_{1A}R$ WT	8.93	0.02	9.09	0.03	7.90	0.03	6.61	0.04
5-$HT_{1A}R$ A365N	8.40	0.06	8.45	0.06	7.23	0.03	5.45	0.03

6. Operational model of bias

Table S4A. Quantification of ligand bias at D_2R using the operational model. The nature of the βarr2 dose response curves for p-tyramine and hordenine do not permit for reliable calculation of bias using the operation model which is indicated by ND in the table.

	G_{oB}		βarr2		G_{oB}/βarr2	bias factor
**compound**	log(τ/KA)	Δlog(τ/KA)	log(τ/KA)	Δlog(τ/KA)	ΔΔlog(τ/KA)	$10^{\Delta\Delta\log(\tau/KA)}$
dopamine	9.25	0.00	7.34	0.00	0.00	1.00
m-tyramine	7.34	-1.91	3.77	-3.57	1.66	45.39
p-tyramine	4.88	-4.37	ND	ND	ND	ND
hordenine	4.77	-4.48	ND	ND	ND	ND
(R)-5-OH-DPAT	8.61	-0.64	4.30	-3.08	2.44	274.16
(S)-5-OH-DPAT	10.61	1.36	8.65	1.31	0.05	1.12
(R)-7-OH-DPAT	9.94	0.69	7.82	0.48	0.21	1.61
(S)-7-OH-DPAT	8.25	-1.01	5.24	-2.10	1.09	12.27

Table S4B. Quantification of ligand bias for dopamine at the D_2R and corresponding mutants using the operational model.

	G_{oB}		βarr2		G_{oB}/βarr2	bias factor
**compound**	log(τ/KA)	Δlog(τ/KA)	log(τ/KA)	Δlog(τ/KA)	ΔΔlog(τ/KA)	$10^{\Delta\Delta\log(\tau/KA)}$
WT D_2R	9.11	0.00	7.21	0.00	0.00	1.00
H393F D_2R	6.68	-2.42	3.32	-3.90	1.47	30.20
H393N D_2R	7.66	-1.45	5.69	-1.53	0.08	1.20

Table S4C. Quantification of ligand bias for (R)-7-OH DPAT at the D_2R and corresponding mutants using the operational model.

	G_{oB}		βarr2		G_{oB}/βarr2	bias factor
**compound**	log(τ/KA)	Δlog(τ/KA)	log(τ/KA)	Δlog(τ/KA)	ΔΔlog(τ/KA)	$10^{\Delta\Delta\log(\tau/KA)}$
WT D_2R	9.84	0.00	7.97	0.00	0.00	1.00
H393F D_2R	8.32	-1.52	3.47	-4.50	2.98	957.19
H393N D_2R	9.60	-0.24	7.54	-0.44	0.20	1.58

Table S4D. Quantification of ligand bias for (S)-5-OH DPAT at the D_2R and corresponding mutants using the operational model.

	G_{oB}		βarr2		G_{oB}/βarr2	bias factor
**compound**	log(τ/KA)	Δlog(τ/KA)	log(τ/KA)	Δlog(τ/KA)	ΔΔlog(τ/KA)	$10^{\Delta\Delta\log(\tau/KA)}$
WT D_2R	10.64	0.00	8.24	0.00	0.00	1.00
H393F D_2R	9.32	-1.32	3.08	-5.16	3.84	6902.40
H393N D_2R	10.94	0.30	8.85	0.61	-0.31	0.49

Table S4E. Quantification of ligand bias for serotonin at the 5-HT_{2A}R and corresponding mutants using the operational model.

	G_q		βarr2		G_q/βarr2	bias factor
**compound**	log(τ/KA)	Δlog(τ/KA)	log(τ/KA)	Δlog(τ/KA)	ΔΔlog(τ/KA)	$10^{\Delta\Delta log(\tau /KA)}$
WT 5-HT_{2A}R	9.53	0.00	6.82	0.00	0.00	1.00
N343A 5-HT_{2A}R	7.36	-2.17	3.66	-3.15	0.98	9.64

Table S4F. Quantification of ligand bias for serotonin at the 5-HT_{1A}R and corresponding mutants using the operational model.

	G_{i2}		βarr2		βarr2/G_{i2}	bias factor
**compound**	log(τ/KA)	Δlog(τ/KA)	log(τ/KA)	Δlog(τ/KA)	ΔΔlog(τ/KA)	$10^{\Delta\Delta log(\tau /KA)}$
WT 5-HT_{1A}R	7.98	0.00	5.88	0.00	0.00	1.00
H365N 5-HT_{1A}R	7.14	-0.84	5.82	-0.07	0.77	5.89

Table S4G. Quantification of ligand bias for serotonin at the 5-HT_{1A}R and corresponding mutants using the operational model.

	G_{oB}		βarr2		βarr2/G_{oB}	bias factor
**compound**	log(τ/KA)	Δlog(τ/KA)	log(τ/KA)	Δlog(τ/KA)	ΔΔlog(τ/KA)	$10^{\Delta\Delta log(\tau /KA)}$
WT 5-HT_{1A}R	8.93	0.00	5.88	0.00	0.00	1.00
H365N 5-HT_{1A}R	8.40	-0.54	5.82	-0.07	0.47	2.94

7. Conformational variability of ligand binding

Typically, GPCR-ligand complexes obtained by X-ray crystallography reveal only one binding mode. In our study, enhanced molecular dynamics simulation shows that dopamine and analogues often adopt different binding modes within the orthosteric binding pocket. In particular, the energetic plot for m-tyramine (Figure 1L in main manuscript) predicts two distinct binding modes which involve an aromatic ring rotation. The energetic barrier between both binding modes is less than 1.5 kcal/mol. This makes it possible to observe both modes in unbiased simulations (Figure S3A). Such diversity in binding modes is not surprising and captured in several X-ray structures (Table S5). An example similar to m-tyramine is shown for the ovine cyclooxygenase-1 in complex with meloxicam (PDB 4O1Z).

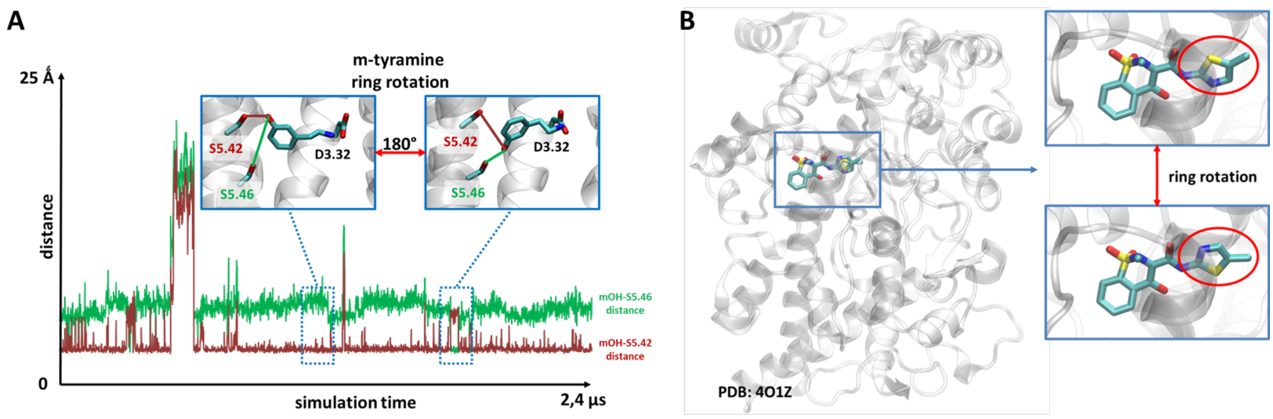

Figure S3. Conformational variability of ligand binding. (A) aromatic ring rotation in m-tyramine and (B) example of a crystallized ring rotation showing cyclooxygenase-1 in complex with meloxicam (PDB 4O1Z)

Table S5. Ligand flips observed in X-ray crystal structures*

PDB ID	chain	residue ID	Resolution [Å]
1EXX	A	450	1.67
1H1R	A	1298	2.00
1XDD	A	401	2.20
2C5N	A	1297	2.10
2C5O	A	1297	2.10
2F71	A	608	1.55
2R3I	A	501	1.28
2R3P	A	501	1.66
2VIP	A	1247	1.72
2VWX	A	1889	1.65
2VWY	A	1889	1.65
2VWZ	A	1889	1.65
2VX1	A	1889	1.65
2X9F	A	1889	1.75
2XNB	A	1299	1.85
3EJJ	A	365	1.80
3O0I	A	237	1.47
3P4V	A	300	2.00
3S9S	A	1	2.55
3SI4	H	1	1.27
4ANW	A	1189	2.31
4EK6	A	301	1.52
4EK8	A	301	1.70
4FTT	A	301	2.30
4IWV	A	503	2.10
4JS3	A	403	2.00
4L2L	A	702	1.65
4O1Z	A	807	2.40

*This list has been kindly provided by Gydo van Zundert, Bijvoet Center for Biomolecular Research, Utrecht University, the Netherlands

8. Chirality-driven binding mode of (R)- and (S)-DPATs

A deeper structural analysis helps clarify why the S-enantiomer binds in an inverted position that allows only for TM5 contacts compared to the corresponding R-enantiomer of the 7-OH-DPAT (Figure S4A-B). A common feature of ligands in aminergic GPCRs is that the protonated nitrogen faces D3.32. In this position the two N-propyl substituents are directed to TM7. We find that a steric requirement for DPAT binding is that the chiral center (red asterisk, Figure S4) of the DPAT scaffold points the smaller substituent, namely the hydrogen (highlighted in red in Figure S4) to the same direction as the bulky N-propyl substituents. This structural arrangement forces the aromatic OH group of (S)-7-OH-DPAT to extend to the bottom of the binding pocket allowing only for TM5 interaction (Figure S4A, right). Our data shows that forming exclusively TM5 interactions is linked to G protein bias. Due to the same steric requirements, the 7-OH group of the R-enantiomer is restricted to point towards the top of the binding pocket. In turn, this binding mode promotes TM5/TM6 contacts (Figure S4B, right) that favors βarr2 recruitment and balanced signaling. The same observation is true for the 5-OH-DPATs. Here, the R-enantiomer points the OH-group down whereas the S-enantiomers orientates it to the top of the binding pocket due to described steric requirements.

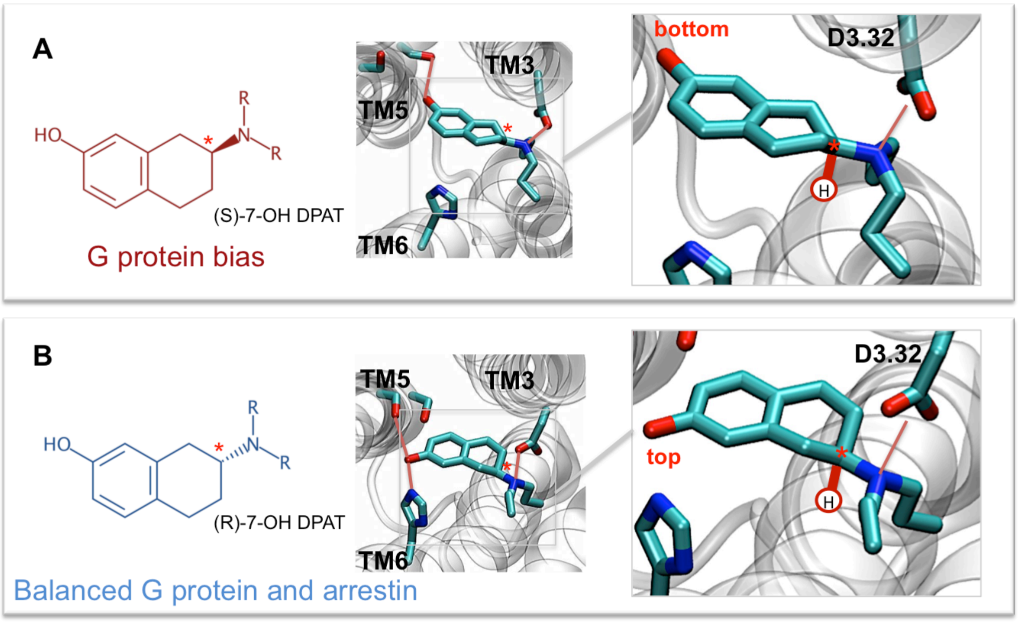

Figure S4. Chirality induces inverted binding modes for 7-OH-DPATs.

9. Ligand interactions with the extracellular loop 2 (ECL2)

Table S6. D_2R MD simulations predict no ECL2 engagement for our set of compounds. Contacts have been computed over preferred binding modes extracted from metadynamics and are defined as distance < 4Å between centre of the ligand and the centre of Ile184. Preferred binding modes have been extracted according to detected energetic wells in Figure 1 and 2.

Compound	Frequency of Ile184 contacts [%]
Dopamine	15
p-tyramine	9
m-tyramine	0
(R)-7-OH-DPAT	0
(S)-7-OH-DPAT	1

10. Contact heatmaps of ligand binding at energetic minimum

Contact heatmaps for ligand binding at energetic minimum provides a general overview of polar contacts between the studied ligands and the D_2R (Figure S5). As expected, we find strong interactions with D3.32 and S5.42 for all studied ligands. In contrast, polar interactions with H6.55 are preferentially observed for ligands with an unbiased dopamine-like coupling profile for G proteins and βarr2. Furthermore, the contact heatmap reveals additional contacts with residues located in TM7 which are only found for dopamine, p- and m-tyramine but not for any of the DPAT derivatives. The reason is that 5-OH-DPATs extend hydrophobic aliphatic substituents (di-propyl groups) towards TM7 which hamper the formation of polar contacts. Nevertheless, we can conclude that these polar contacts do not contribute to the coupling specificity as they are existent in compounds with a dopamine-like coupling profile (i.e. dopamine) as well as with a G protein biased profile (i.e. p-tyramine).

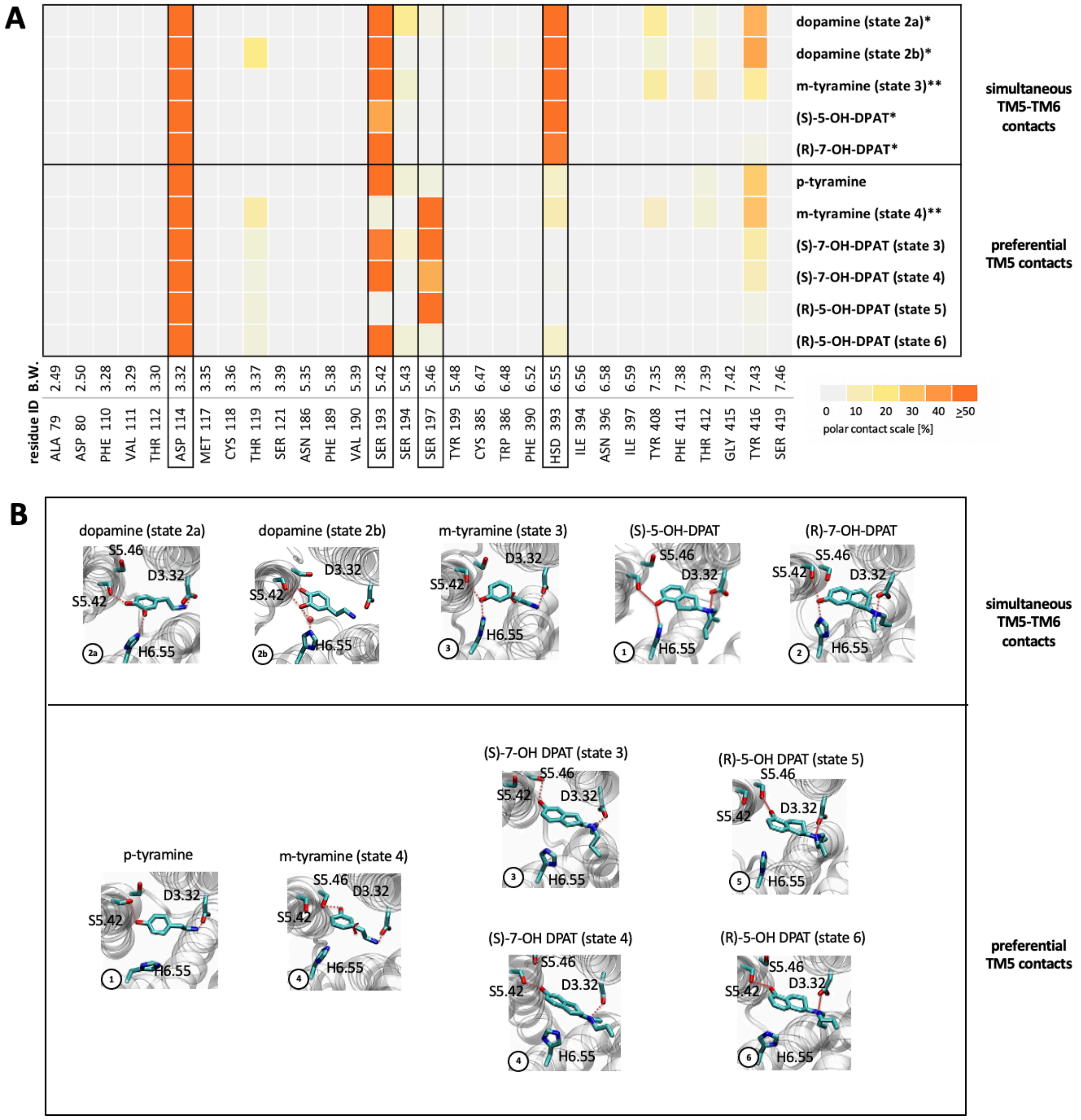

Figure S5. Contact maps for polar ligand-D_2R interactions. (A) Contact maps highlighting key interactions in the orthosteric binding site at an energetic minimum. Metadynamics have been filtered for ligand binding poses that correspond to their energetic minimum. Polar contacts including direct and water-mediated interactions have been computed across the filtered simulations using the GetContacts pipeline on GPCRmd[9]. Colors correspond to the contact frequency expressed as percentage of formed contacts across simulation frames. Compounds with an unbiased dopamine-like coupling profile (highlighted with *) are typically characterized by simultaneous TM5 and TM6 interactions. Interestingly, m-tyramine (highlighted with **) co-exists in two binding modes with simultaneous TM5-TM6 (state 3) or exclusive TM5 (state 4). See main text for discussion on this topic. (B) Low energetic binding modes detected in metadynamics.

11. Binding signature of hordenine and its coupling outcome

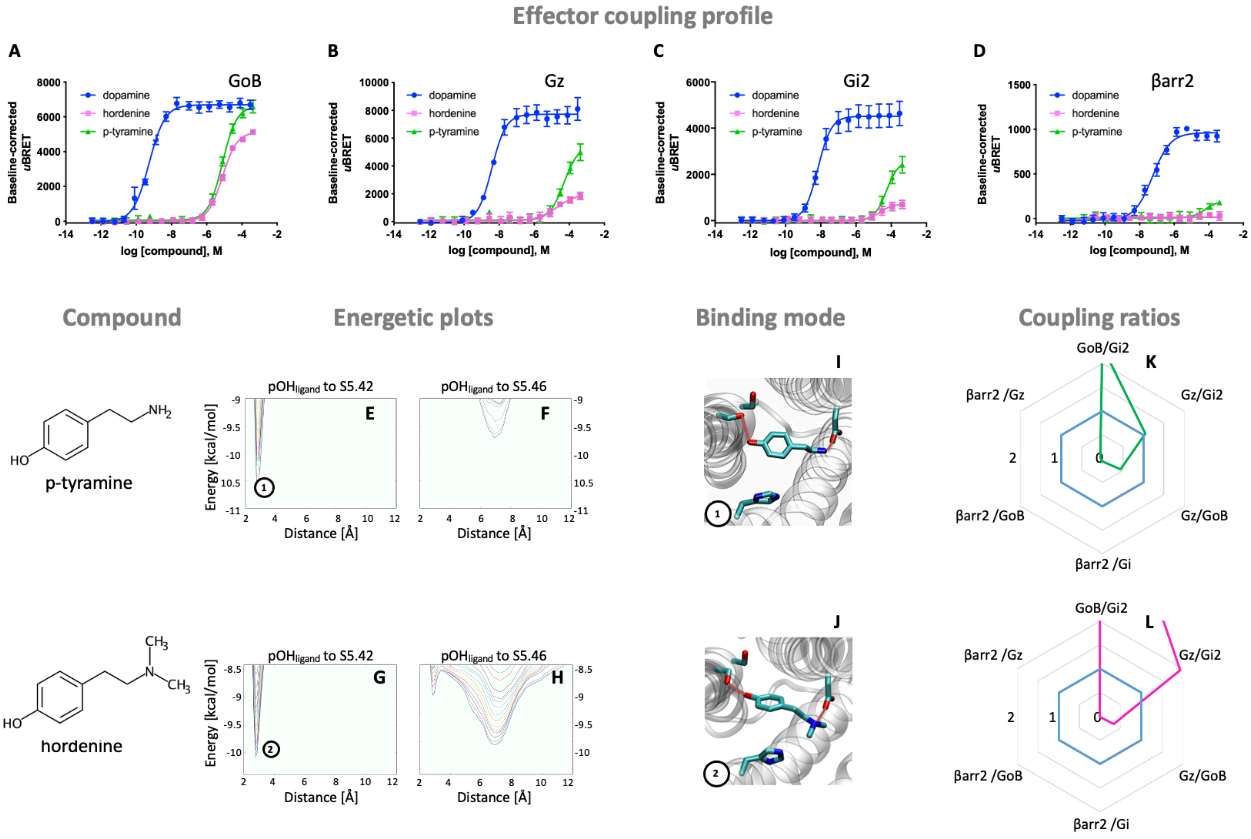

Figure S6. Coupling profile and binding profile of hordenine versus p-tyramine. The corresponding chemical structures are depicted on the left. **A-D:** Concentration-response curves of dopamine (blue), p-tyramine (green) and hordenine (pink) -induced coupling of the D_2R to G_{oB}, G_z, G_{i2} and β-arrestin 2 (βarr2). For corresponding pEC50 and Emax values see Table S3. **E-H:** Energetic plots of ligand binding obtained by metadynamics using as metrics the distance of the p-OH groups to S5.42 and S5.46. An energetic well at ~2.8 Å indicates a favorable distance for binding contacts with the corresponding residue. To ensure convergence of binding energetics, we monitored free energy profiles along simulation by plotting the profile every 20000 deposited Gaussians (graphs shown in different colors). **I-J:** Representative structures of the binding mode corresponding to the energetic wells identified in the energetic plots. **K-L:** Coupling ratios were approximated using the area under the curve (AUC) and its ratio for individual signaling effectors (e.g. βarr2 vs G_z, βarr2 vs G_{oB} etc.). Note that to eliminate the observational bias linked to differences within different recruitment assays (e.g. βarr2 vs G_i), we use dopamine as internal standard for analyzing AUCs. The coupling profile of the reference compound dopamine is denoted by a coupling ratio of 1 for all pathway combinations and highlighted in all plots as a blue line. Preferential or disfavoured coupling are indicated by ratios > 1 or < 1, respectively. Dose-response curves were generated using data obtained from 3 independent experiments.

12. Sequence analysis across dopamine, serotonin and adrenergic receptors

A complete sequence analysis of positions involved in neurotransmitter binding for dopamine, serotonin and adrenaline receptors (26 GPCRs) provides an overview of their conservation (3.32, 5.42, 5.46 and 6.55, Figure S7A). Overall, it appears that most diversity is found in the serotonin receptor family compared to dopamine and adrenergic receptors. Across the studied set, position 3.32 and 5.42 are highly conserved whereas more differences are found in position S5.46 and 6.55. We envisage that such variation determines the coupling and signaling outcome for different subtype receptors within a family and beyond. For instance, differences in the polarity of position 6.55 will likely impact βarr coupling and in turn receptor

internalization. It is tempting to speculate that the group of α1 adrenergic receptors with a nonpolar residue (L,M6.55) is likely to have an innately reduced arrestin coupling compared to the β adrenergic receptors with a conserved polar residue in position 6.55 (N6.55). A similar tendency is expected for the 5-HT_{1A}R (A6.55) in comparison to the group of 5-HT_2, 5-HT_4, 5-HT_6 and 5-HT_7 (N6.55). This goes along with our finding that the 5-HT_{1A}R can be converted into a 5-HT_2-like receptor with enhanced βarr coupling properties by introducing a polar residue into position 6.55 (A6.55N, Figure 5). At times, substitution can be also polar-to-polar as seen in the dopaminergic receptor family (H6.55N). Such polar-to-polar substitutions should be able to mostly preserve the key interaction with the neurotransmitter and thus receptor coupling properties which is supported by experimental validation of the D_2R H6.55N mutant (Figure 3).

Finally, also natural genetic variations of these positions will alter the functional outcome of a specific receptor. Analysis of healthy individuals (data extracted from the GPCRdb database[10,11] and the Exome Aggregation Consortium[12]) reveal primarily rare events (frequency < 1%) (Figure S7B) with the highest occurrence in the serotonin receptor family when compared to dopamine and adrenergic receptors (Figure S7B). Nevertheless, genetic variances within all three studied families seem to be well tolerated as they are observed in healthy individuals.

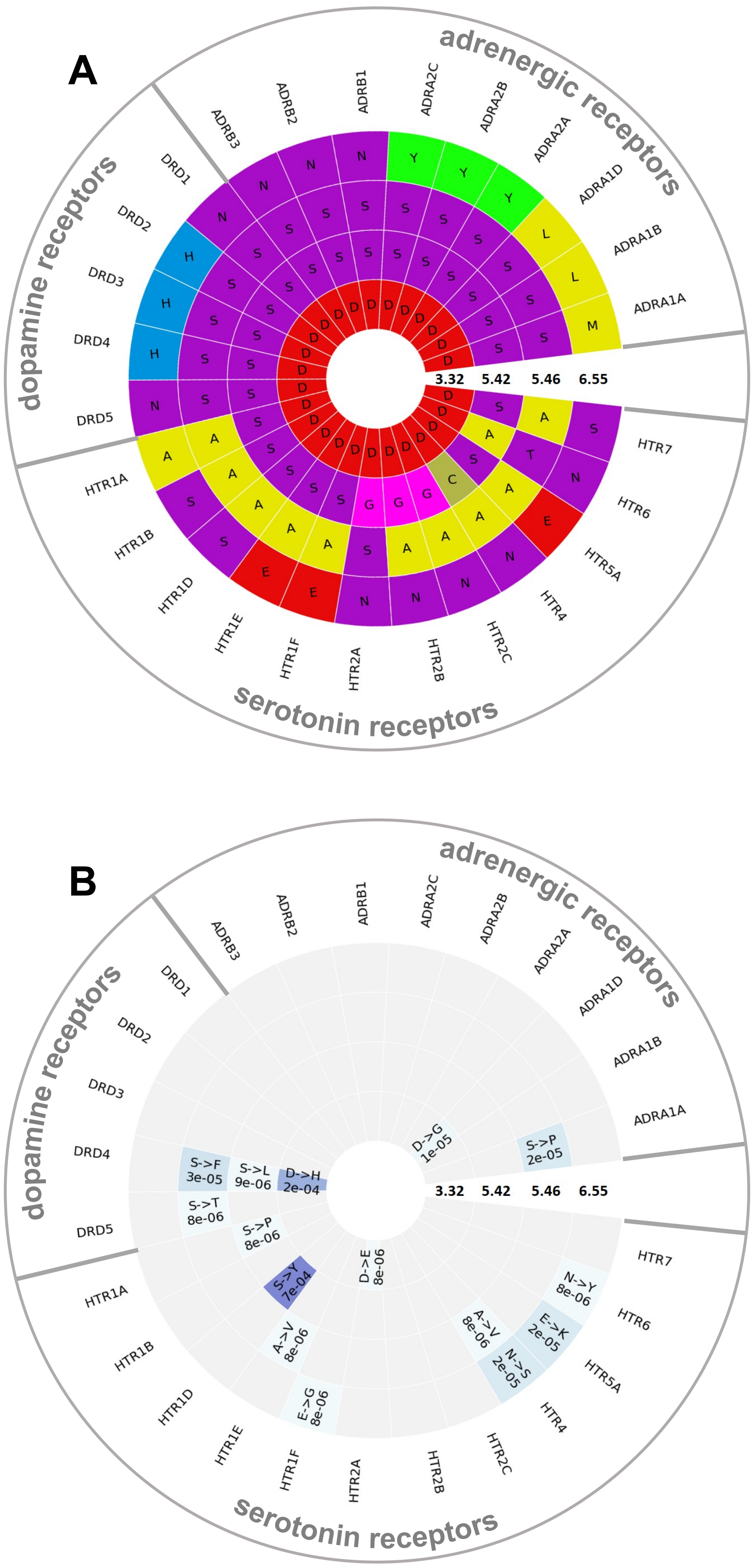

Figure S7. Sequence conservation and natural genetic variance in key position for neurotransmitter binding across dopamine, serotonin and adrenergic receptors. (A) Sequence alignment and conservation of key positions forming the salt bridge (3.32) and polar contacts in TM5 (5.42, 5.46) and TM6 (6.55). (B) Natural genetic variance indicating the type of substitution and frequency extracted from the GPCRdb database[10,11] and the Exome Aggregation Consortium.[12]

13. Experimental Procedures

Radar graph generation. Radar graphs were produced using the area under the dose response curves (AUC; calculated using GraphPad Prism 6 software). For every compound tested, the calculated pathway-specific AUCs were divided by one another (e.g., AUC $_{GoB}$/AUC G_{i2}, AUC βarr2/AUC G_{oB}, etc.) resulting in "relative AUCs". To eliminate the influence of system (i.e. different coupling sensitivity of partners), we use dopamine as internal standard to obtain standardized coupling ratios for all compounds (Table S7). Relative AUCs for dopamine were used as reference values to which corresponding relative AUCs for other compounds were normalized (e.g., [AUC βarr2/AUC G_{oB}] (R)-5-OH-DPAT / [AUC βarr2/AUC G_{oB}] Dopamine). These normalized values, referred to as "normalized relative AUCs", were plotted as radar graphs. Normalization of AUCs for mutant receptor radar graph generation was conducted using an identical approach. However, relative AUCs for the mutant receptors were always normalized to the corresponding relative AUCs for the WT receptor. For hD_2R mutants (for which multiple ligands were tested), the relative AUCs for dopamine for the WT receptor were used as reference values to which all other corresponding relative AUCs were normalized.

Table S7. Quantification of the coupling ratios using area under the curves (AUC) from dose-response curves. AUCs for each pathway were obtained using PRISM 7.0 (**Pathway-specific AUCs**). The relative coupling ratios for each compound (**Relative AUCs**) were calculated by dividing corresponding pathway-specific AUCs (e.g. G_{oB} /G_{i2}). Then, normalized coupling ratios were obtained from relative coupling ratios using dopamine as a reference compound (**Normalized Relative AUCs**).

	**Pathway-specific AUCs**<br>AUC per pathway			
	**G_{i2}**	**G_{oB}**	**G_z**	**βarr2+GRK2**
dopamine	14068	24603	23675	1494
m-tyramine	6799	17205	12475	338
p-tyramine	1497	6180	2639	9
hordenine	874	9033	2858	1
(R)-5-OH-DPAT	7979	20850	15346	467
(S)-5-OH-DPAT	16099	30297	27013	1805
(R)-7-OH-DPAT	13994	27420	24451	1434
(S)-7-OH-DPAT	8516	19773	15659	623

	**Relative AUCs**<br>$\text{relative AUC} = \frac{\text{AUC}_{\text{pathway1}}}{\text{AUC}_{\text{pathway2}}}$					
	**G_{oB} /G_{i2}**	**G_z /G_{i2}**	**G_z /G_{oB}**	**βarr2 /G_{i2}**	**βarr2 /G_{oB}**	**βarr2 /G_z**
dopamine	1.75	1.68	0.96	0.11	0.06	0.06
m-tyramine	2.53	1.83	0.73	0.05	0.02	0.03
p-tyramine	4.13	1.76	0.43	0.01	0.00	0.00
hordenine	10.34	3.27	0.32	0.00	0.00	0.00
(R)-5-OH-DPAT	2.61	1.92	0.74	0.06	0.02	0.03
(S)-5-OH-DPAT	1.88	1.68	0.89	0.11	0.06	0.07
(R)-7-OH-DPAT	1.96	1.75	0.89	0.10	0.05	0.06
(S)-7-OH-DPAT	2.32	1.84	0.79	0.07	0.03	0.04

	**Normalized Relative AUCs**<br>$\text{normalized relative AUC} = \frac{\text{relative AUC}_{\text{compound x}}}{\text{relative AUC}_{\text{dopamine}}}$					
	**G_{oB} /G_{i2}**	**G_z /G_{i2}**	**G_z /G_{oB}**	**βarr2 /G_{i2}**	**βarr2 /G_{oB}**	**βarr2 /G_z**
dopamine	1	1	1	1	1	1
m-tyramine	1.45	1.09	0.75	0.47	0.32	0.43
p-tyramine	2.36	1.05	0.44	0.06	0.02	0.05
hordenine	5.91	1.94	0.33	0.01	0.00	0.01
(R)-5-OH-DPAT	1.49	1.14	0.76	0.55	0.37	0.48
(S)-5-OH-DPAT	1.08	1.00	0.93	1.06	0.98	1.06
(R)-7-OH-DPAT	1.12	1.04	0.93	0.96	0.86	0.93
(S)-7-OH-DPAT	1.33	1.09	0.82	0.69	0.52	0.63

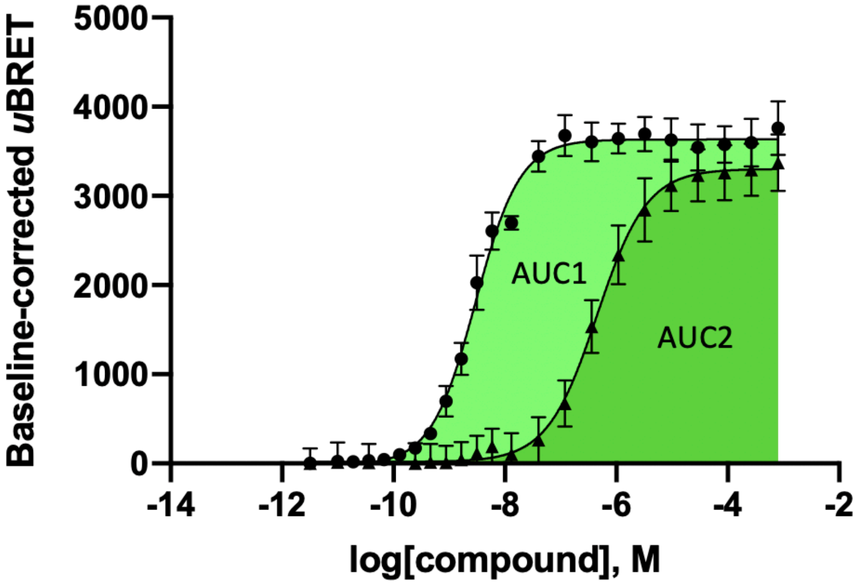

Figure S8. Schematic representation of the area under the curve (AUC) for dose response curves calculated using GraphPad Prism 6 software. AUC1 from dose response curves 1 is greater than the AUC2 from dose response curves 2 reflecting its higher potency and efficacy.

References

[1] R. Woodward, C. Coley, S. Daniell, L. H. Naylor, P. G. Strange, *J. Neurochem.* **1996**, *66*, 394–402.

[2] J. C. Fowler, S. Bhattacharya, J. D. Urban, N. Vaidehi, R. B. Mailman, *Mol. Pharmacol.* **2012**, *81*, 820–831.

[3] C. Coley, R. Woodward, A. M. Johansson, P. G. Strange, L. H. Naylor, *J. Neurochem.* **2000**, *74*, 358–366.

[4] B. L. Wiens, C. S. Nelson, K. A. Neve, *Mol. Pharmacol.* **1998**, *444*, 435–444.

[5] B. A. Cox, R. A. Henningsen, T. Spanoyannis, T. L. Neve, K. A. Neve, *J. Neurochem.* **1992**, *59*, 627–635.

[6] R. C. Kling, N. Tschammer, H. Lanig, T. Clark, P. Gmeiner, *PLoS One* **2014**, *9*, 1–10.

[7] N. Tschammer, S. Bollinger, T. Kenakin, P. Gmeiner, T. Park, N. C. T. K, *Mol. Pharmacol.* **2011**, *79*, 575–585.

[8] W. Humphrey, A. Dalke, K. Schulten, *J. Molec. Graph.* **1996**, *14*, 33–38.

[9] I. Rodríguez-Espigares, M. Torrens-Fontanals, J. K. S. Tiemann, D. Aranda-García, J. M. Ramírez-Anguita, T. M. Stepniewski, N. Worp, A. Varela-Rial, A. Morales-Pastor, B. Medel-Lacruz, G. Pándy-Szekeres, E. Mayol, T. Giorgino, J. Carlsson, X. Deupi, S. Filipek, M. Filizola, J. C. Gómez-Tamayo, A. Gonzalez, H. Gutiérrez-de-Terán, M. Jiménez-Rosés, W. Jespers, J. Kapla, G. Khelashvili, P. Kolb, D. Latek, M. Marti-Solano, P. Matricon, M. T. Matsoukas, P. Miszta, M. Olivella, L. Perez-Benito, D. Provasi, S. Ríos, I. R. Torrecillas, J. Sallander, A. Sztyler, S. Vasile, H. Weinstein, U. Zachariae, P. W. Hildebrand, G. De Fabritiis, F. Sanz, D. E. Gloriam, A. Cordomi, R. Guixà-González, J. Selent, *Nat. Methods* **2020**, *17*, 777–787.

[10] G. Pándy-Szekeres, C. Munk, T. M. Tsonkov, S. Mordalski, K. Harpsøe, A. S. Hauser, A. J. Bojarski, D. E. Gloriam, *Nucleic Acids Res.* **2018**, *46*, D440–D446.

[11] A. S. Hauser, S. Chavali, I. Masuko, L. J. Jahn, K. A. Martemyanov, D. E. Gloriam, M. M. Babu, *Cell* **2018**, *172*, 1–14.

[12] M. Lek, K. J. Karczewski, E. V. Minikel, K. E. Samocha, E. Banks, T. Fennell, A. H. O'Donnell-Luria, J. S. Ware, A. J. Hill, B. B. Cummings, T. Tukiainen, D. P. Birnbaum, J. A. Kosmicki, L. E. Duncan, K. Estrada, F. Zhao, J. Zou, E. Pierce-Hoffman, J. Berghout, D. N. Cooper, N. Deflaux, M. DePristo, R. Do, J. Flannick, M. Fromer, L. Gauthier, J. Goldstein, N. Gupta, D. Howrigan, A. Kiezun, M. I. Kurki, A. L. Moonshine, P. Natarajan, L. Orozco, G. M. Peloso, R. Poplin, M. A. Rivas, V. Ruano-Rubio, S. A. Rose, D. M. Ruderfer, K. Shakir, P. D. Stenson, C. Stevens, B. P. Thomas, G. Tiao, M. T. Tusie-Luna, B. Weisburd, H. H. Won, D. Yu, D. M. Altshuler, D. Ardissino, M. Boehnke, J. Danesh, S. Donnelly, R. Elosua, J. C. Florez, S. B. Gabriel, G. Getz, S. J. Glatt, C. M.

Hultman, S. Kathiresan, M. Laakso, S. McCarroll, M. I. McCarthy, D. McGovern, R. McPherson, B. M. Neale, A. Palotie, S. M. Purcell, D. Saleheen, J. M. Scharf, P. Sklar, P. F. Sullivan, J. Tuomilehto, M. T. Tsuang, H. C. Watkins, J. G. Wilson, M. J. Daly, D. G. MacArthur, *Nature* **2016**, *536*, 285–291.
